# Abstracts from the Sixth Bioelectronic Medicine Summit: Neurotechnologies for Individuals and Communities

**DOI:** 10.1186/s42234-025-00182-9

**Published:** 2025-10-21

◦ Sponsor note: Publication of this supplement was funded by the Institute of Bioelectronic Medicine, Feinstein Institutes for Medical Research.

◦ Co-hosted by the Feinstein Institutes for Medical Research/Northwell Health and the University of Minnesota.

◦ Sponsored by Blackrock Neurotech (Diamond Plus), Spark Biomedical Inc (Diamond), iota Biosciences, Inc. (powered by Astellas) (Diamond), SetPoint Medical (Silver), Hofstra University (Silver), Focused Ultrasound Foundation (Bronze), Tucker-Davis Technologies (Bronze), Cognito Therapeutics (Bronze), Neuromodec (Media Sponsor), Tivic Health (Media Sponsor), Christina Epicure (Media Sponsor), Ridgewood Savings Bank (Media Sponsor).

◦ With collaboration from Bioelectronic Medicine (journal), Center for Bioelectronic Medicine, Molecular Medicine, BioMed Central, and BMC Springer Nature.

## **P2** Selective peripheral nerve stimulation: computational models to the rescue

### Nicolò Rossetti^1^, Philipp Schnepel^1^, Vojkan Mihajlović^1^, Weiguo Song^2^, Timir Datta-Chaudhuri^2^, Stavros Zanos^2^

#### ^1^IMEC, Eindhoven, NLD; ^2^Institute of Bioelectronic Medicine, Feinstein Institutes for Medical Research and Northwell Health, Manhasset, NY, USA

##### **Correspondence:** Nicolò Rossetti (nicolo.rossetti@imec.nl); Stavros Zanos (szanos@northwell.edu)

*Bioelectronic Medicine 2025,* **11(1):**P2

The promise of bioelectronic medicine to provide neuromodulation-based alternative to drug-based treatments of chronic diseases relies on the ability of neurostimulation devices to achieve selective stimulation of neural targets in order to maximize the efficacy of the therapy while minimizing off-target stimulation side effects. Selective stimulation of peripheral nerves requires usage of multiple contact cuff electrodes (MCEs) and novel stimulation waveforms leveraging multiple stimulation sources simultaneously. In order to understand the effects and optimize stimulation parameters for these complex paradigms, acute animal experiments with MCE interfaces are usually performed. However, such neural recordings do not provide access to single-fiber or small fiber population (e.g., single-fascicle) responses while access to animals and experimental protocols can impose time limits on exhaustive parameter explorations. Realistic computational models based on 3D anatomies of reconstructed nerves and cuff geometries, combined with accurate electrophysiological fiber models are a powerful tool to shed light onto the mechanism of action of new waveforms, and can be leveraged to optimize stimulation protocols.

Here we describe how computational models can be combined with data from in vivo experiments to help explain the mechanism of action of a new intermittent interferential current stimulation (i^2^CS) paradigm, applied to the pig Vagus nerve via an MCE. I^2^CS delivers high-frequency interferential stimulation (in the order of 20kHz, having an amplitude modulated signal at a few kHz) through short pulses of (sub-)ms duration, rendering only half of the beat. We obtained simulated neural responses from the computational model that leverages the ASCENT pipeline, which correlate with physiological readouts (muscle activation and breathing rate) obtained during in vivo stimulation. Analysis of single-fiber responses revealed that i^2^CS induces activation delays at the focus of interference, which is consistent with a thresholding activation mechanism. The pulse duration can be leveraged to tune the activation delay in a precise way compared to less-predictable continuous interferential stimulation. We also show how increased activation threshold at the location of interference can be leveraged to increase stimulation selectivity for a given function. Finally, we propose how computational models can be combined with physiological data from in vivo experiments to estimate the unknown nerve anatomy and how this could be used to optimize stimulation protocols.

Keywords: bioelectronic medicine, vagus nerve, selective peripheral nerve stimulation, computational modeling

## **P3** Long term use of non-invasive auricular neurostimulation in combination with ustekinumab to maintain remission of pediatric Crohn’s disease: a case report

### Kristine Pascuma^1^, Jillian Charyn^1^, Kristen Lacey^1^, Benjamin Sahn^1,2^

#### ^1^Cohen Children’s Medical Center, Northwell Health, New Hyde Park, NY, USA; ^2^Feinstein Institutes for Medical Research, Manhasset, NY, USA

##### **Correspondence:** Kristine Pascuma (kpullen@northwell.edu); Benjamin Sahn (bsahn@northwell.edu)

*Bioelectronic Medicine 2025,* **11(1):**P3

Introduction: Transcutaneous electrical stimulation of the cymba concha targets the auricular branch of the vagus nerve (taVNS) to activate the cholinergic anti-inflammatory pathway. This non-invasive form of nerve stimulation is an investigational therapy to treat immune mediated inflammatory diseases (IMIDs) with effectiveness reported in short term clinical trials with small sample sizes. Long-term taVNS for this purpose is not well reported. We review a case of successful long-term use in a child with Crohn’s disease.

Case Report: Patient SS presented at age 4 years old in 2013 with abdominal pain and bloody stools. Crohn’s disease involving the duodenum and colon was found. She was treated with mesalamine (2013-2014) unsuccessfully, then azathioprine (2014-2017) leading to a clinical and biochemical remission. Due to patient concerns about long term risk of immunomodulatory medication, azathioprine was discontinued. She returned to clinical care in 2019 with abdominal pain, fecal calprotectin 333 ug/g and colonoscopy finding active Crohn’s disease of her duodenum and right colon. She enrolled in a 16-week clinical trial using taVNS. The TENS 7000 stimulation device was used with parameters of a normal pulse wave, 20 Hz frequency, and 300 µs pulse width for 5 minutes twice daily. At the end of 16 weeks, her abdominal pain resolved with overall no symptoms reported. Her fecal calprotectin normalized to 59 ug/g. She then continued taVNS as her sole long term Crohn’s disease maintenance therapy. In the first year, adherence to the taVNS stimulation varied between zero, once, or twice daily use.

After 1 year of taVNS, her symptoms remained resolved, however her calprotectin increased to previous levels 388 ug/g. Repeat endoscopy found mildly active duodenitis and much improved but not resolved macroscopic disease in the right colon compared to colonoscopy before taVNS therapy was initiated. In 2021, given active disease persisting, ustekinumab (Interleukin 12/23 antagonist) was initiated and taVNS use was increased to a consistent 5 minutes twice daily with adherence. Two years later in 2023, with durable clinical remission achieved, reevaluation of her disease activity identified a completely normalized fecal calprotectin 14 ug/g and complete mucosal healing (endoscopic and histologic healing) of her duodenum and colon. She continues this combination of ustekinumab and taVNS with excellent growth, nutritional status, and clinical remission of her Crohn’s disease to date.

Discussion: Transcutaneous electrical neurostimulation of the auricular cymba concha is being investigated for treatment of intestinal IMIDs such as Crohn’s disease and ulcerative colitis. Its excellent safety profile allows taVNS to be considered as a potential monotherapy or in combination with a conventional pharmacological therapy as we report here. A limitation of taVNS as compared to an implanted device is the potential for non-adherence, which can impact therapy success. Its mechanistic effect through vagus nerve mediated anti-inflammatory signaling and potentially other neural circuits requires further investigation.

*The subject/proxies described in the abstract gave consent permissions for this case report.

Keywords: Neuromodulation, vagus nerve, pediatrics, Crohn's disease

## **P4** The effect of Baroreflex Activation Therapy on inflammatory biomarkers and heart rate variability in patients with heart failure with reduced ejection fraction

### Alexandra Bekiaridou^1,2^, Kristie Coleman^3^, Nhan Nguyen^3^, Seth Wilks^4^, Alfio Carroccio^3^, Stefanos Zafeiropoulos^2^, Dimitrios Varrias^3^, Haisam Ismail^3^, Marcin Kowalski^3^, Miguel Alvarez^3^, Sirish Vullaganti^3^, Thedoros Zanos^2^, Stavros Zanos^1,2^, Stavros Mountantonakis^3^

#### ^1^Elmezzi Graduate School of Molecular Medicine, Northwell Health, Manhasset, NY, USA; ^2^The Feinstein Institutes, Northwell Health, Manhasset, NY, USA; ^3^Lenox Hill Hospital, Northwell Health, NY, USA; ^4^CVRx, Minneapolis, MN, USA

##### **Correspondence:** Alexandra Bekiaridou (abekiaridou@northwell.edu); Stavros Mountantonakis (smountanto@northwell.edu)

*Bioelectronic Medicine 2025,* **11(1):**P4

Background: Recent advances in understanding the pathophysiology of heart failure with reduced ejection fraction (HFrEF) have highlighted the critical role of sympathovagal imbalance and inflammation. In this context, Barostim^TM^ has emerged as the first FDA-approved neuromodulation device for Baroreflex Activation Therapy (BAT), specifically targeting this sympathovagal imbalance in HFrEF patients and potentially providing anti-inflammatory benefits.

Objective: To assess the effect of BAT on inflammatory biomarkers and heart rate variability (HRV) to provide physiological insight into the mechanism of action.

Methods: Seven HFrEF patients were implanted with the BAT device. Assessment was performed at baseline before the implantation of Barostim (pre-BAT) and 6 months post maximum uptitration (post-BAT). Outcomes included 6-minute walking test (6MWT), arrhythmia burden, heart failure hospitalizations, HRV with time- and frequency- domain analysis, and quality of life (QoL) assessed by the Minnesota Living with Heart Failure Questionnaire (MLHFQ) and circulating inflammatory biomarkers.

Results: The mean age of the patients was 69.04±8.74 and 2 of them were females. Their mean Left Ventricular Ejection Fraction was 24.55±11.58. Regarding NYHA Functional Class, 4 patients were Class II, and 3 patients were Class III. BAT led to a 94% decrease of HF hospitalizations 12-months after implantation [12-months before: 3 (0-4), 12-months after: 0 (0-0), p= 0.007]. Additionally, patients reported improvement in QoL, as measured by MLHFQ with a reported difference in the overall score of −42 [Overall score at baseline: 63 (57-71.5) and after 6-months: 21 (19.5-21.75), p=0.01]. TNF-a and IFN-γ were decreased post-BAT (TNF-a at baseline: 2.15±0.47 and 6 months post-BAT: 1.55 ±0.35, p= 0.03; IFN-γ at baseline: 7.82±4.3 and 6 months post-BAT: 3.14±1.45, p=0.03). HRV time-domain measure showed that SDNN was increased after BAT [baseline: 92 (89-105), 6-months after: 101 (91-109), p=0.03] (Fig. 1).

Conclusion: BAT in HFrEF patients led to a decrease in TNF-a and IFN-γ, highlighting a novel potential mechanism of BAT. An ongoing expanded study will allow us to assess clinical phenotypes of patients with differential potential of response to treatment. These findings suggest that Barostim may exert its beneficial effects by not only correcting autonomic dysregulation but also by attenuating inflammatory pathways, offering a dual mechanism of action that enhances its efficacy in HFrEF treatment.

Keywords: heart failure with reduced ejection fraction, neuromodulation, barostimulation

**Fig. 1 (abstract P4).**
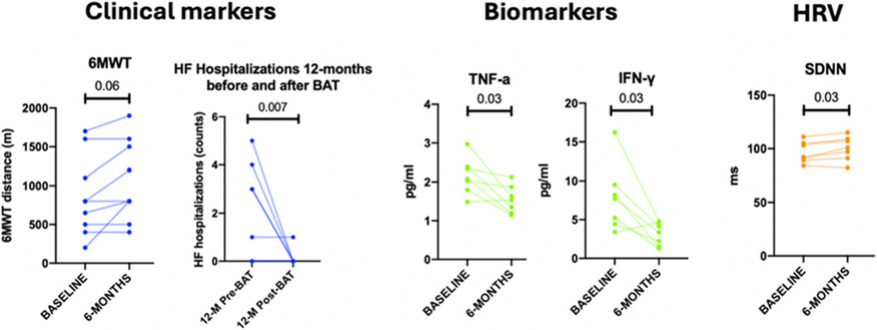
See text for description

## **P5** Impact of sex in effects of splenic focused ultrasound on experimental pulmonary hypertension

### Alexandra Bekiaridou^1,2^, Ibrahim Mughrabi^1^, Ethan Paliwoda^1^, Haris Iqbal^1^, Nicolas Rios^1^, Stavros Zanos^1,2^

#### ^1^Elmezzi Graduate School of Molecular Medicine, Northwell Health, Manhasset, NY, USA; ^2^The Feinstein Institutes, Northwell Health, Manhasset, NY, USA

##### **Correspondence:** Alexandra Bekiaridou (abekiaridou@northwell.edu); Stavros Zanos (szanos@northwell.edu)

*Bioelectronic Medicine 2025,* **11(1):**P5

Background: Pulmonary Arterial Hypertension affects predominantly women, but with reduced severity. We recently discovered that noninvasive splenic focused ultrasound (FUS) suppresses inflammatory cell infiltration in the lung and improves experimental PH in male rats. The effect of FUS on females is unknown.

Methods: PH was induced using Monocrotaline (MCT, 60 mg/kg SQ) in male and female rats. Animals received daily FUS or sham stimulation, for 14 days and, at the end of treatment, they were subjected to right heart catheterization. Lung flow cytometry (FC) was performed.

Results: RVSP was significantly increased in PH animals of both sexes (PH males, PHm, 53.88±2.04 vs healthy males, Hm, 25.43±3.01, p<0.0001; PH females, PHf, 37.00±3.93 vs healthy females, Hf, 20.44±0.57, p=0.0005). FUS reduced RVSP compared to sham (PHm+FUS 43.32±1.35 vs PHm+sham 53.88±2.04, p=0.02; PHf+FUS 25.34±1.74 vs PHf+sham 37.00±3.93, p=0.01). Lung CD8+ cells were increased in PH animals (PHm 134.2±11.08 vs Hm 100.6±12.23 p=ns, and PHf 153.9±15.17 vs Hf 92.92±18.30 p=0.02). FUS reduced CD8+ cells (PHm+sFUS 79.68±8.89 vs PHm+sham 134.2±11.08 p=0.03; PHf+FUS 71.9±7.41 vs PHf+sham 153.9±15.17 p<0.01). Lung CD68+ macrophages were increased in PH (PHm 37.54±3.46 vs healthy males 16.81±0.93, p<0.01; females PH 37.66±6.19 vs healthy females 16.96±1.11, p=0.001). SFUS reduced lung macrophages (PH males+FUS 25.12±0.93 vs PHm+sham 37.54±3.46, p=0.01 and PH females+FUS 23.42±0.95 vs PH females+FUS- 37.66±6.19, p=0.03). CD43HiHis48Int-Lo anti-inflammatory non-classical monocytes were increased in PH (PH males 120.3±26.96 vs healthy males 68.4±16.19 p ns, and PHf 117.5±18.02 vs Hf 96.80±11.53 p=0.99). SFUS increased the population of non-classical monocytes (PHm+FUS 220.5±41.27 vs PHm+sham 120.3±26.96, p=ns and PHf+FUS 262.8±41.23 vs PHf+sham 117.5±18.02, p=0.01).

Conclusion: Female rats develop a less severe phenotype of the disease induced by MCT. In females, FUS essentially normalizes RVSP and inflammatory cell infiltration in the lung.

Keywords: Focused Ultrasound, Pulmonary Hypertension

## **P6** Methodological framework for analyzing enteric nervous system activity in awake rats

### Amparo Güemes^1^, Alexander J. Boys^2,3^, George G. Malliaras^1^, Róisín M. Owens^3^

#### ^1^Department of Engineering, University of Cambridge, Cambridge, UK; ^2^Thayer School of Engineering, Dartmouth College, Hanover, NH, USA; ^3^Department of Chemical Engineering & Biotechnology, University of Cambridge, Cambridge, UK

##### **Correspondence:** Amparo Güemes (ag2239@cam.ac.uk)

*Bioelectronic Medicine 2025,* **11(1):**P6

Introduction: The enteric nervous system (ENS) regulates gastrointestinal functions and influences overall health through local neural processing distributed throughout the gut (1). Conventional neural recording devices face significant challenges in maintaining stable, conformal contact with the highly elastic and dynamically moving tissues of the gastrointestinal (GI) tract. Consequently, in vivo electrophysiological recordings of the ENS in freely moving subjects have been largely absent. Here, we detail a methodology for analyzing colonic electrophysiology signals in response to stress and feeding, using a state-of-the-art bioelectronic device based on thin film technology implanted in freely moving rats.

Methods: Novel thin film probes were implanted in the colonic wall of Sprague Dawley rats (n=4) in close juxtaposition with enteric nerves. Data collection occurred on Days 1, 8, and 12. Since gut electrophysiological recordings have not yet been conducted in freely moving animals, we opted to investigate the impact of two conditions: an acute anxiolytic environment (open-field test) and the response to a food stimulus on gut-derived electrical signals. As a result, we captured complex multi-frequency electrophysiological responses in response to stress and feeding with Nutella®. Custom Python software was used to process the data. Signals at 10KHz were referenced to a common baseline to reduce artifacts. The data were divided into frequency bands using bandpass filters (2,3): 0–0.2 Hz for slow-wave activity (interstitial cells of Cajal), 0.2–1 Hz for smooth muscle slow rhythms, 1–5 Hz for faster smooth muscle activity, 5–300 Hz for skeletal muscle movements, and 300–2000 Hz for high-frequency neural activity. The Power Spectral Density (PSD) was calculated using the Welch's method. Normalized power for each band was computed to ensure fair comparisons across bandwidths over time.

Results and Discussion: In our investigation of gut activity under stress, we observed a high initial electrophysiological power across frequency bands during the first 8 minutes on Day 1, which declined over time in the open field. This trend diminished by Day 8 and Day 12. Summarizing the overall power across frequency bands during the first 15 minutes each day revealed a clear downward trend, likely reflecting stress acclimatization. For the study under meal intake, we observed consistent upward trends in power across all frequency bands following feeding on all days, particularly in the slow-wave and neural activity bands, aligning with the known increase in colonic activity during food intake. Variations in feeding behavior and intake likely explain the variability observed between animals. These findings highlight the potential for real-time monitoring of gut neural activity in response to stress and feeding.

Conclusion: This study presents the first analysis of in vivo electrophysiology recordings of neural signaling in the ENS in awake animals, offering insights into gut neural dynamics during stress and feeding and setting the stage for future exploration of the gut-brain connection.

Keywords: Electrophysiology, enteric nervous system, bioelectronics, multi-frequency analysis

**References**

1. Cryan, J. F. et al. The Microbiota-Gut-Brain Axis. Physiol Rev 99, 1877–2013 (2019).
2. Chen, J.-D. Spectral analysis of electrogastrogram and its clinical significance. http://www.wjgnet.com/ 2, 9–11 (1996).
3. Ding, F., Guo, R., Cui, Z.-Y., Hu, H. & Zhao, G. Clinical application and research progress of extracellular slow wave recording in the gastrointestinal tract. World J Gastrointest Surg 14, 544 (2022).

## **P7** Island-based pixel detection for mapping tyrosine hydroxylase-positive fiber distribution in vagal fascicles

### Jinxuan Cang^1,2^, Viktor Toth^1,2^, Naveen Jayaprakash^1,3^, Avantika Vardhan^1,2^, Todd J. Levy^1,2^, Siyar Bahadir^1,3^, Khaled Qanud^1,3^, Nafiseh Saleknezhad^1,3^, Nicole Carpentiere^1,3^, Ariadni Markantonaki Kouletaki^1,2^, Demetra Kostaridis^1,2^, Theofilos Kanavos^1,2^, Effrosyni Birbas^1,2^, Stavros Zanos^1,3,4^, Theodoros P. Zanos^1,2,4^

#### ^1^Northwell Health, New Hyde Park, NY, USA; ^2^Institute of Health System Science, Feinstein Institutes for Medical Research, Northwell Health, Manhasset, NY, USA; ^3^Feinstein Institutes for Medical Research, Northwell Health, Manhasset, NY, USA; ^4^Donald and Barbara Zucker School of Medicine at Hofstra/Northwell, Northwell Health, Hempstead, NY, USA

##### **Correspondence:** Jinxuan Cang (jcang@northwell.edu); Theodoros P. Zanos (tzanos@northwell.edu)

*Bioelectronic Medicine 2025,* **11(1):**P7

The vagus nerve innervates multiple organs. By electrically stimulating the vagus nerve, organ functions can be modulated for therapeutic purposes. The human vagus nerve consists of over 100,000 fibers, and these fibers are organized into fascicles. The spatial arrangement of fibers within fascicles and their target organs are poorly understood. Better understanding of fiber spatial arrangements informs electrode design and stimulation waveform parameters.

Traditionally, the vagus nerve has been regarded as a major trunk of the parasympathetic nervous system. Nonetheless, recent studies have found tyrosine hydroxylase (TH)-positive fibers in the vagus nerve. TH is a well-known catecholamine-synthetic enzyme in the production pathways of dopamine and norepinephrine. The presence of excitatory neurotransmitters strongly suggests sympathetic functions of the vagus nerve; however, their distribution within vagal fascicles is not elucidated.

To better understand the TH-positive fiber distribution, and their co-localization with myelinated and unmyelinated fibers in fascicles, we employ fluorescence microscopy combined with pixel-island detection algorithms. fluorescence microscopy targets both structural proteins (neurofilament and myelin basic protein) and enzymatic proteins (including TH). To capture multiple stains simultaneously, a monochromatic camera records each fluorescent emission wavelength as a separate channel. An edge detection algorithm then isolates each fascicle, using only the neurofilament (NF) and myelin basic protein (MBP) to estimate the fascicle positions.

Due to tissue artifacts, the fluorescence intensity histograms for TH images are bimodal. The lower peak represents the background noise, while the upper peak corresponds to TH+ signal. To filter out background noise, histologists use manual annotations to designate true-negative and true-positive pixels. The annotations are used to derive a false-positive and a true-positive intensity threshold, where the true-positive intensity is always higher than that of false-positive.

TH+ islands are identified using a connected-pixel method. Initially, pixels exceeding the true-positive threshold are designated as the nucleation points for TH+ islands. Adjacent pixels in a 4-point pattern are then evaluated against the false-positive threshold. Pixels above the false-positive threshold are classified into the same island. This pixel-connection process continues, propagating outward from the nucleation points, until no adjacent pixels are above the false-positive threshold. This approach differs from our previously published NF+ island detection. Compared to the NF algorithm, the connected-pixel method is stain-agnostic and does not require network training.

When multiple stains are present, channel images are segmented separately to avoid detection interference. After compensating for coordinate shifts, any remaining overlaps are attributed to co-expression of proteins. NF+/TH+ islands are identified by locating the nearest NF+ islands to each TH+ island based on centroid distances. Overlapping areas are re-assigned to NF+ islands and are removed from TH+ islands to avoid double counting.

The island-based detection method streamlines co-stain recognition by completely de-coupling from the stain segmentations. It offers flexibility in adapting to co-expression criteria as biological understanding evolves. Finally, it provides accurate detection and assists in the determination of TH+ fiber distribution in vagal fascicles.

Keywords: Vagus nerve anatomy, vagus nerve stimulation, fluorescence microscopy, fluorescence multiplexing, tyrosine hydroxylase, connected-pixel segmentation, instance segmentation, pixel-island detection, co-expression detection.

## **P8** Lumbosacral spinal cord epidural stimulation reduces blood pressure instability by attenuating spasms of lower limb muscles

### Pawan Sharma^1^, Siqi Wang^2^, Claudia Angeli^1,3,4^, Gail Forrest^1,4^, Enrico Rejc^1^, Sara Wagers^2^, Maxwell Boakye^2^, Susan Harkema^1^

#### ^1^Tim and Caroline Reynolds Center for Spinal Stimulation, Kessler Foundation, NJ, USA; ^2^Kentucky Spinal Cord Injury Research Center, Louisville, KY, USA; ^3^Department of Bioengineering, University of Louisville, Louisville, KY, USA; ^4^PM & R, Rutgers New Jersey Medical School, Rutgers, The State University of New Jersey, Newark, NJ, USA

##### **Correspondence:** Pawan Sharma (PSharma@kesslerfoundation.org); Susan Harkema (SHarkema@KesslerFoundation.org)

*Bioelectronic Medicine 2025,* **11(1):**P8

Introduction: Cardiovascular dysfunction after cervical spinal cord injury (SCI) results in blood pressure instability from persistent hypotension, episodes of high blood pressure (autonomic dysfunction) and orthostatic hypotension. We reported that continuous lumbosacral spinal cord epidural stimulation targeted for cardiovascular function (CV-scES) stabilizes the systolic blood pressure during daily activities and ameliorates orthostatic hypotension. We observed additional rapid increases in the systolic blood pressure when individuals had sustained bouts of unintentional muscle activation during spasms. We hypothesized that epidural stimulation targeted to reduce unintentional muscle activation (M-scES) would prevent the increase in systolic blood pressure.

Methods: Preliminary data was collected from four cervical SCI participants who underwent implantation of a 5-6-5 Medtronic epidural stimulation electrode array over the lumbosacral spinal cord. We first investigated the activation profile of abdominal and lower extremity muscles during spasm events. All participants then underwent spatiotemporal mapping to identify effective anode and cathode selection to prevent unintentional activation of muscle during spasmodic events. While in the relaxed seated position in a wheelchair, hemodynamic data were collected using a three-lead ECG (Finapres Medical Systems, Amsterdam, Netherlands) and beat-to-beat blood pressure recorded from the index finger using photoplethysmography (Finapres Medical Systems, Amsterdam, Netherlands). Simultaneously, we recorded abdominal and lower limb muscle activity using a multi-channel EMG setup. We identified CV-scES parameters that stabilized the blood pressure instability and M-scES parameters that reduced the duration and severity of spasms.

Results: When the initiation of the spasm was recognized, we immediately switched from CV-scES program to M-scES program. We observed significant and early reduction in the unintentional lower limb muscle activity and systolic blood pressure compared to maintaining CV-scES program alone.

Conclusions: Besides sympathetic overactivity, the presented result demonstrates that blood pressure instability following SCI can also occur due to muscle spasms. Specific lumbosacral epidural stimulation strategies targeting blood pressure and lower limb spasms can synergistically reduce blood pressure instability in SCI.

Keywords: Spinal Cord Injury, Epidural Stimulation, Systolic Blood Pressure, Cardiovascular System, Lower Limb Spasms

## **P10** Innovative implantable neurotechnologies to restore the biomimetic control of the lower urinary tract

### A. Giannotti^1^, M. Ceradini^1^, S. Musco^2^, F. Bernini^3^, G. Del Popolo^2^, S. Micera^1,4,5^

#### ^1^BioRobotics Institute, Health Science Interdisciplinary Research center, and Department of Excellence Robotics and AI, Scuola Superiore Sant’Anna, Pisa, ITA; ^2^Careggi University Hospital, florence, ITA; ^3^Life Science Institute, Scuola Superiore Sant’Anna, Pisa, ITA; ^4^Modular implantable neurotechnologies (MINE) Laboratory, Università Vita-Salute San Raffaele & Scuola Superiore Sant’Anna, Milan, ITA; ^5^Translational Neural Engineering Laboratory, neuro-X institute, École Polytechnique Federale de Lausanne (EPfl), Lausanne, CHE

##### **Correspondence:** A. Giannotti (alice.giannotti@santannapisa.it); S. Micera (silvestro.micera@epfl.ch)

*Bioelectronic Medicine 2025,* **11(1):**P10

Restoring urinary function is highly desired by patients with neurogenic lower urinary tract (LUT) dysfunctions, as these impairments severely affect quality of life. Bioelectronic medicine offers a promising alternative to traditional therapies, but current neuromodulation devices lack real-time adaptability, using continuous or intermittent stimulation that often leads to reduced efficacy due to neural adaptation. Closed-loop stimulation paradigms have shown the potential to increase bladder capacity and micturition efficiency, but they require robust, real-time ability to decode bladder fullness.

The study aimed to develop an innovative implantable neurotechnology to restore adaptive, biomimetic control of the LUT using a bidirectional intraneural interface targeting bladder-controlling nerves. Using a large animal model to ensure the translation of experimental results into clinical practice, a decoding algorithm based on intraneural pudendal nerve signals was validated for real-time bladder pressure prediction. A minimally invasive trans-gluteal approach was used to implant 16-channel intra-neural interfaces in the left pudendal nerve of a female anesthetized pig (3 months, 30 kg). A dual-lumen catheter (6-Fr) measured intravesical pressure, while a rectal catheter (10-Fr) monitored abdominal pressure. Detrusor pressure was calculated as the difference between intravesical and abdominal pressure. Intra-neural pudendal nerve signals were recorded during bladder filling with saline solution at 50 ml/min until urination was observed. A custom-developed deep-learning-based algorithm based on a Long Short-Term Memory network was trained on neural signals from a cystometric curve, allocating 80% of the dataset for training and 20% for testing.

The custom-designed deep learning algorithm utilized peak-to-peak amplitude from filtered intraneural pudendal signals (300-1000 Hz range) to estimate detrusor pressure. Each time point utilized approximately 1 second of preceding neural data, indicating the algorithm’s potential for real-time application. The algorithm demonstrated high predictive accuracy, with an average estimated value above the 80^th^ percentile of the true detrusor pressure. Statistical validation of the algorithm was performed using a Pearson correlation coefficient (r = 0.9182) between predicted and observed detrusor pressure values, to assess the predictive accuracy of the deep-learning model. This research demonstrates that intraneural recordings from the pudendal nerve could reliably predict bladder pressure, opening the path for closed-loop neuroprosthethics tailored to patient needs in real-time toward the restoration of a more physiological urination cycle.

Keywords: Neurogenic lower urinary tract dysfunction, Closed-loop neuroprosthesis, Intraneural pudendal nerve recordings, Real-time bladder pressure estimation

## **P11** A high-density, multi-contact slanted electrode array allows selective, intraneural recording and stimulation of the swine vagus nerve

### Alice Giannotti^1^, Weiguo Song^2^, Khaled Qanud^2^, Stavros Zanos^2^

#### ^1^The Biorobotics Institute & Department of Excellence in Roboti*cs *and AI, Sant'Anna School of Advanced Studies, Pisa, ITA; ^2^The Feinstein Institute for Medical Research, Northwell Health, Manhasset, NY, USA

##### **Correspondence:** Alice Giannotti (alice.giannotti@santannapisa.it); Stavros Zanos (szanos@northwell.edu)

*Bioelectronic Medicine 2025,* **11(1):**P11

Vagus nerve stimulation (VNS) is a proven therapy for epilepsy and depression and is under investigation for treating chronic inflammatory diseases such as heart failure, inflammatory bowel disease, and rheumatoid arthritis. Current VNS devices utilize bipolar cuffs that stimulate the entire nerve, ignoring its spatial organization and often causing side effects from unintended fiber activation. High-density, multi-contact slanted electrode arrays (MEAs) may overcome these limitations by enabling selective recording and stimulation of vagus nerve fibers, potentially enhancing VNS efficacy and safety. We tested MEAs in the cervical vagus nerve of anesthetized swine (n=3, ~50kg). MEAs were implanted using a pneumatic insertion pump and paired with a helical cuff electrode for comparison. Physiological responses, such as heart rate (HR), breathing rate (BR), and blood pressure (BP), as well as neural activity, were monitored during baseline and aCer pharmacological baroreflex activation using phenylephrine. Stimulation trains (30 Hz, 200 µs) delivered via the MEA or cuff elicited graded changes in physiological parameters, such as a 10% BR drop, 8% HR reduction, and 3% BP decrease. Stimulation through MEAs required significantly lower currents (200-300 µA) compared to cuffs (1000- 1500 µA) to induce comparable responses. Spontaneous vagus nerve activity was successfully recorded with the MEA but not with the cuff electrode. Neural spikes, consistent with action potentials, were identified from 89 of the 96 MEA contacts, with signal-to-noise ratios (SNR) of 4.42 ± 1.28. Spike sorting revealed single-fiber activity in 74 contacts and two distinct clusters in 28 contacts, demonstrating the MEA’s ability to detect fine neural dynamics. Firing rates were predominantly below 1 Hz, with occasional bursts up to 5 Hz during baroreflex activation. These results highlight the capability of MEAs to monitor dynamic vagal signaling in real-time. Compared to traditional cuff electrodes, the MEA enabled spatially selective stimulation, likely due to its ability to target specific vagal fascicles. This selectivity may reduce side effects by minimizing off-target fiber activation. Additionally, MEAs demonstrated superior recording sensitivity, capturing spontaneous and evoked neural activity that could not be detected with the cuff. The swine vagus nerve serves as a relevant model for human applications due to its multifascicular structure. Our findings suggest that MEAs hold promise for improving VNS by enabling organ-specific modulation and real-+me monitoring of vagal signaling. Future studies will focus on chronic implants in awake, freely moving animals to validate these results under physiological conditions. With their ability to selectively interact with nerve fibers, high-density MEAs may enhance the safety, efficacy, and adaptability of VNS therapies for various clinical applications.

Keywords: Vagus nerve stimulation, high-density intraneural prosthesis, large animal model, organ-specific modulation

## **P12** Development of polymer-based packaging for chronic wireless neuromodulation implants in mice

### Jason Wong, Ibrahim Mughrabi, Tyler Hepler, Mohamed Elgohary, Michael Recine, Timir Datta-Chaudhuri

#### Institute for Bioelectronic Medicine, Feinstein Institutes for Medical Research, Northwell Health, Manhasset, NY, USA

##### **Correspondence:** Jason Wong (jwong26@northwell.edu); Timir Datta-Chaudhuri (tdatta@northwell.edu)

*Bioelectronic Medicine 2025,* **11(1):**P12

Clinical active implantable medical devices have strict packaging requirements to protect both the electronics and the patient during long-term implantation in the human body. Although metals, glass, and ceramics provide reliable hermetic sealing, their fabrication is complex and costly, so polymers present an attractive, lower-cost choice for preclinical studies where resources are more constrained and long-term durability is less critical. However, designing implants for small animal research remains challenging due to limitations on mass and volume. Various parameters including choice of polymer, material thicknesses, and processing methods must be considered to create an implantable system suitable for chronic use in mice.

Two implantable package designs were designed and manufactured for use with a wireless neuromodulation system and tested on the benchtop and *in vivo*. One design relied on complete encapsulation, embedding electronics in biocompatible polymers, while the other used an enclosure, sealing electronics within a polymer shell. Packaging performance was evaluated using two test methods. Bench testing was performed by soaking devices in saline at an elevated temperature to accelerate moisture- and ion-ingress related failure. *In vivo* testing involved chronic implantation in C57BL/6 mice. In both tests, the packaged wireless neuromodulation systems were regularly checked to confirm functionality of all device features such as electrical stimulation, ECG recording, wireless communication, and wireless charging.

The enclosure-style packaging was found to be preferred over the encapsulation packaging due to the shorter preparation times, lower mass, and significantly improved circuit accessibility for troubleshooting, failure analysis, and reuse. Median lifetimes for enclosure-packaged systems exceeded 30 days across all testing indicating that this approach can support chronic mouse experiments of approximately 1 month in length.

Keywords: implantable, packaging, semi-hermetic, neuromodulation, small animal research

## **P13** A pipeline for building scalable 3D cytoarchitecture models from cleared intact whole human ganglia and peripheral nerve samples

### Siyar Bahadir^1^, Todd Levy^1^, Naveen Jayaprakash^1^, Khaled Qanud^1^, Parisa Saleknezhad^1^, Avantika Vardhan^1^, Zeinab Nassrallah^2^, Mary Barbe^3^, Eric Chang^1^, Theodoros P. Zanos^1,2^, Stavros Zanos^1,2^

#### ^1^Feinstein Institutes for Medical Research, Institute of Bioelectronic Medicine - Manhasset, NY, USA; ^2^Donald and Barbara Zucker School of Medicine at Hofstra/Northwell, Northwell Health, Hempstead, NY, USA; ^3^Temple University, Philadelphia, PA, USA

##### **Correspondence:** Siyar Bahadir (sbahadir@northwell.edu); Stavros Zanos (szanos@northwell.edu)

*Bioelectronic Medicine 2025,* **11(1):**P13

Targeted stimulation of human peripheral nerves and ganglia is an important goal in the fields of neuromodulation and bioelectronic medicine. While significant progress has been made in the field of connectomics, similar efforts for peripheral nerves in the human nervous system face significant methodological hurdles. Existing techniques, such as 3D micro-CT imaging and single-fiber mapping through serial sectioning and immunohistochemistry staining, have not provided an established method to image these structures intact in 3D space with sufficient resolution to build scalable single-fiber/single-cell 3D cytoarchitecture models.

A major challenge has been the difficulty in tracking the long-range projections of axons in peripheral nerves. Unlike central nervous system neurons, which are more arborized and for which most automated and semi-automated neural tracing algorithms are designed, peripheral nerve axons are long, straight, and tightly packed. Protocols and tracing algorithms optimized for detecting branches and synapses over short length scales do not perform well for the peripheral nervous system's morphology.

In this work, we present 3D cytoarchitecture reconstructions obtained from a cleared human nodose ganglion with proximal vagus nerve samples and human dorsal root ganglion with a cleared and expanded spinal nerve sample. We have developed a robust semi-automated fiber-tracking algorithm specifically designed to track the long, straight, and tightly packed axons of the peripheral nervous system. This algorithm overcomes previous limitations by efficiently tracing axonal pathways.

Our pipeline represents a significant advance in visualizing and creating parametrizable single-cell/single-fiber models of human peripheral ganglia and nerves within their 3D spatial context. By overcoming methodological challenges, we provide a foundation for building highly detailed connectomic maps of human peripheral neural tissues. This work enhances the understanding of the in-situ anatomy of cells and fibers within human peripheral nerves and ganglia, which is crucial for targeted stimulation applications in neuromodulation and bioelectronic medicine.

Keywords: human nodose ganglion, peripheral nerves, computational anatomy, light sheet microscopy, expansion microscopy

## **P14** Data loss mitigation techniques for wireless streaming of neural recording data from an implanted device

### Mohamed Elgohary, Michael Recine, Jason Wong, Timir Datta-Chaudhuri

#### The Feinstein Institutes for Medical Research, Northwell Health, Manhasset, NY, USA

##### **Correspondence:** Mohamed Elgohary (melgohary@northwell.edu); Timir Datta-Chaudhuri (tdatta@northwell.edu)

*Bioelectronic Medicine 2025,* **11(1):**P14

Reliable wireless streaming of real-time neural signals from implantable devices is critical for conducting high-quality studies that advance our understanding of the nervous system. Neural signals often contain subtle features, which can be critical for accurate analysis. Consequently, any lapse in data continuity could obscure meaningful insights or lead to misleading conclusions. Neural data requires high sampling rates and resolution that rivals the highest quality audio requirements, and though existing wireless standards like Bluetooth Low Energy are known to be robust and well-optimized for applications such as streaming to wireless earbuds—they may not be sufficient for low-power implantable devices where the roles of transmitter and receiver are reversed. The unique challenges for implantable devices in small animal research that stem from power and size constraints as well as signal attenuation by the body, result in less-than-ideal radio operation.

Within this context, we investigate strategies aimed at mitigating data losses and ensuring continuous, high-quality wireless data transfer from the implant. Our exploration includes evaluating variations of retransmission protocols, data interleaving, data redundancy, and power modulation when constructing and transmitting radio packets. Furthermore, when the alternative is data loss, we consider temporarily relaxing the requirement of a high sampling rate and dynamically reduce resolution when the wireless channel experiences pronounced interference, ensuring that the data stream continues uninterrupted. We assess performance based on the ability of the strategies to prevent data loss, with priority given to preventing gaps in time while also minimizing power consumption, as excessive energy consumption reduces the operating lifetime of the implant. Testing was performed in a benchtop environment where implementations of the different strategies were repeated to average out environmental effects. We found that the best performing strategies are a combination of data interleaving and some form of redundancy, alongside a higher radio output power.

Keywords: Implantable, neuromodulation, small animal research, neural, recording, wireless, real-time, streaming, chronic, radio, packet loss, mitigation, data loss

## **P15** α7nAChR agonism blunts nuclear HMGB1 translocation in dorsal root ganglia neurons

### Timothy S. Morgan^1^, Huan Yang^1^, Serena Petruzzelli^1^, Saher Chaudhry^1^, Sangeeta S. Chavan^1,2,3^, Kevin J. Tracey^1,2,3^

#### ^1^Institute for Bioelectronic Medicine, Feinstein Institutes for Medical Research, Manhasset, NY, USA; ^2^Elmezzi Graduate School of Molecular Medicine, Feinstein Institutes for Medical Research, Northwell Health, Manhasset, NY, USA; ^3^Donald and Barbara Zucker School of Medicine at Hofstra/Northwell Health, Hempstead, NY, USA

##### **Correspondence:** Timothy S. Morgan (tmorgan9@northwell.edu); Kevin J. Tracey (kjtracey@northwell.edu)

*Bioelectronic Medicine 2025,* **11(1):**P15

High Mobility Group Box 1 (HMGB1) is a nuclear protein that maintains chromatin organization and nuclear stability under normal conditions. However, during cellular stress, HMGB1 translocates from the nucleus to the cytoplasm and extracellular space, where it acts as a damage-associated molecular pattern (DAMP), initiating inflammation through activation of TLR4 and RAGE on proximal immune cells, microglia, and sensory neurons. The α7 nicotinic acetylcholine receptor (α7nAChR) is a critical mediator of the cholinergic anti-inflammatory pathway, where it facilitates the anti-inflammatory effects of choline acetyltransferase positive T cells on systemic immune cells. Since activation of α7nAChR has been shown to suppress HMGB1 release and cytokine production in immune cells, this suggests that it may similarly regulate HMGB1 translocation in sensory neurons. In this study, we investigated whether activation of α7nAChR in dorsal root ganglia (DRG) sensory neurons could blunt capsaicin-induced HMGB1 translocation from the nucleus to the cytoplasm. To test this, DRGs were harvested from C57BL/6 mice, cultured for 48 hours, and treated with vehicle, capsaicin (5 µM), or capsaicin plus, α7nAChR agonist, GTS-21 (10 µM). After treatment, DRGs were stained with 4′,6-diamidino-2-phenylindole (DAPI) to label nuclei, neuronal nuclei marker (NeuN) and immunostained for HMGB1. DRGs were then imaged to quantify the total cytoplasmic HMGB1+ area and the percentage of neurons with cytoplasmic HMGB1 in each condition. Capsaicin treatment alone significantly increased both the percentage of DRG neurons with cytoplasmic HMGB1 localization (60% ± 3.06 vs. 17.3% ± 2.67; P < 0.0001) and the HMGB1-positive cytoplasmic area (433.14 ± 35.37 µm² vs. 173.99 ± 14.21 µm²; P < 0.0001) compared to vehicle-treated controls. However, co-treatment with GTS-21 significantly reduced both measures of HMGB1 translocation, with a lower percentage of neurons displaying cytoplasmic HMGB1 compared to capsaicin alone (Capsaicin + GTS-21: 36% ± 1.15 vs. Capsaicin: 60% ± 3.06; P = 0.0011) and a decreased HMGB1-positive cytoplasmic area (Capsaicin + GTS-21: 201.19 ± 16.43 µm² vs. Capsaicin: 433.14 ± 35.37 µm²; P < 0.0001). These results demonstrate that α7nAChR activation suppresses capsaicin-induced nuclear-to-cytoplasmic translocation of HMGB1 in DRG neurons. Vagus nerve stimulation has been shown to activate the cholinergic anti-inflammatory pathway, increasing acetylcholine (ACh) levels, both through direct release from vagal efferents and by stimulating ChAT+ T cells in circulation. Thus, the cholinergic anti-inflammatory pathway may regulate inflammatory and nociceptive responses by attenuating the release of neuronal HMGB1 through α7nAChR-mediated mechanism.

Keywords: HMGB1, α7 nicotinic acetylcholine receptor, cholinergic anti-inflammatory pathway, neuroinflammation, dorsal root ganglia

## **P16** Focused ultrasound of the spleen mitigates traumatic hemorrhage via Chat+ T-lymphocytes and A7 nicotinic acetylcholine receptors

### Carlos E. Bravo-Iñiguez^1^, Kevin J. Tracey^1^, Sangeeta S. Chavan^1^, Jared M. Huston^1,2,3^

#### ^1^Institute of Bioelectronic Medicine, Feinstein Institutes for Medical Research/Northwell Health, Manhasset, NY, USA; ^2^Department of Surgery, Northwell Health, Manhasset, NY, USA; ^3^Department of Science Education, Zucker School of Medicine at Hofstra/Northwell, Hempstead, NY, USA

##### **Correspondence:** Carlos E. Bravo-Iñiguez (cbravoiniguez@northwell.edu); Jared M. Huston (jhuston@northwell.edu)

*Bioelectronic Medicine 2025,* **11(1):**P16

Introduction: Trauma is the leading cause of death among Americans aged 1 to 45, with uncontrolled hemorrhage being the most common preventable factor in these fatalities. While direct pressure or tourniquets can help control extremity bleeding, surgical intervention is required to manage non-compressible truncal hemorrhage. Unfortunately, there are few systemic therapies available to promote hemostasis. Electrical stimulation of the vagus nerve (VNS) promotes faster clotting to minimize traumatic hemorrhage in mice, relying on a pathway that involves ChAT+ T-lymphocytes in the spleen and ⍺7 nicotinic acetylcholine receptors (⍺7nAChR) on circulating platelets. Focused ultrasound (FUS) stimulation of spleen decreases traumatic hemorrhage. Using a murine tail transection hemorrhage model, we examine whether ChAT+ T lymphocytes or ⍺7nAChR are essential for hemostasis after FUS.

Methods: Adult male wild-type (C57BL6), conditional T-lymphocyte knockout (CD4-ChAT-/-), or ⍺7nAChR knockout mice (⍺7KO) are anesthetized (ketamine/xylazine), placed in the right lateral decubitus position, and the spleen is identified by surface anatomy. The ultrasound probe (Sonic Concepts, H101) is placed on shaved skin with ultrasound gel and aimed at the splenic hilum. The function generator (33120A, Keysight Technologies) and power amplifier (350L RF, Electronics and Innovations) deliver 1 min of stimulation (1.1 MHz, 200 mV per pulse, 150 burst cycles, 500 μs burst period), followed by 30 s of rest, and then 1 min of stimulation. Sham stimulated animals receive FUS over the right quadriceps. Animal tails are warmed in water (37.1°C, 5 min), transected 2 mm from the tip, and bled into water (37.1°C) until hemorrhage stops for at least 10 s. Hemorrhage duration is recorded as bleeding time.

Results: Compared with sham stimulation, FUS significantly reduces bleeding time in wild-type mice (Sham = 110.5 ± 7.7 s vs. FUS = 72.9 ± 6.6 s, mean ± SEM, n = 10, p < 0.01, t-test). Compared with sham stimulation, FUS fails to decrease bleeding time in CD4-ChAT-/- mice (Sham = 117.5 ± 11.3 s vs. PFUS = 91.0 ± 14.9 s, n = 5, p=ns), or ⍺7KO mice (Sham = 125.7 ± 9.22 s vs. PFUS = 127.4 ± 9.54 s, n = 8, p=ns).

Conclusions: FUS stimulation of the spleen requires CD4-ChAT+ T-lymphocytes and ⍺7nAChR to decrease hemorrhage. Further clinical research is needed to explore its potential in trauma, surgical bleeding, and bleeding disorders.

Keywords: bleeding, trauma, focused ultrasound, spleen

## **P17** Trans-spinal focused ultrasound reduces mechanical sensitivity and suppresses spinal microglia activation in rats with chronic constriction injury

### Weiguo Song^1^, Alice Giannotti^2^, Alexandra Bekiaridou^3^, Ona Bloom^1^, Stavros Zanos^1^

#### ^1^Feinstein Institutes for Medical Research/Northwell Health, Manhasset, NY, USA; ^2^The BioRobotics Institute, Department of Excellence in Robotics and AI, Scuola Superiore Sant’Anna, Pisa, ITA; ^3^Elmezzi Graduate School of Molecular Medicine, Feinstein Institutes for Medical Research, Manhasset, NY, USA

##### **Correspondence:** Weiguo Song (wsong2@northwell.edu); Stavros Zanos (szanos@northwell.edu)

*Bioelectronic Medicine 2025*, **11(1):**P17

Since published at: https://bioelecmed.biomedcentral.com/articles/10.1186/s42234-025-00170-z

Keywords: trans-spinal focused ultrasound stimulation, noninvasive neuromodulation, Von Frey threshold, pain, flow cytometry, microglia

## **P18** Single cell transcriptional profiling of PBMC after transcutaneous auricular nerve stimulation

### Aisling Tynan^1^, Timothy Morgan^1^, Alejandro Torres^1^, Isabella Mirro^1^, Carlos Bravo Iniguez^1^, Okito Hashimoto^1^, Kevin J. Tracey^1,2,3^, Sangeeta S. Chavan^1,2,3^

#### ^1^Institute for Bioelectronic Medicine, Feinstein Institutes for Medical Research/Northwell Health, Manhasset, NY, USA; ^2^Donald and Barbara Zucker School of Medicine at Hofstra/Northwell Health, Hempstead, NY, USA; ^3^The Elmezzi Graduate School of Molecular Medicine, Feinstein Institutes for Medical Research, Manhasset, NY, USA

##### **Correspondence:** Aisling Tynan (atynan@northwell.edu); Sangeeta S. Chavan (schavan@northwell.edu)

*Bioelectronic Medicine 2025,* **11(1):**P18

Neural circuits regulate cytokine production to prevent excessive inflammation. Transcutaneous auricular nerve stimulation (tANS), a non-invasive neuromodulation technique, has gained attention for its therapeutic potential in inflammatory and autoimmune disorders. However, the molecular and cellular mechanisms underlying its effects remain insufficiently characterized. Here, we investigate the effects of tANS on human peripheral blood mononuclear cells (PBMCs) using single-cell RNA sequencing (scRNA-seq) and cytokine profiling.

tANS was performed on the cymba concha using surface electrodes connected to a transcutaneous electrical nerve stimulation (TENS) device. Healthy participants (*n*=3) underwent a 5-minute stimulation (pulse width: 200 μs; pulse frequency: 20 Hz; current: adjusted to sensory threshold). Whole blood was collected pre- and 2 hours post-tANS for an ex vivo *lipopolysaccharide* (LPS) challenge. PBMCs were isolated for scRNA-seq, and plasma was assayed for inflammatory cytokines.

We analyzed a total of 32,430 cells across four conditions: Pre-0LPS, Pre-10LPS, Post-0LPS, and Post-10LPS. No significant changes were observed in the overall proportions of monocytes, T cells, NK cells, or B cells pre- and post-tANS. However, differential gene expression analysis revealed a significant change in monocyte population following tANS. A significant downregulation of proinflammatory transcription factors *NFKB1, FOSB,* and *JUN *and LPS -induced proinflammatory cytokine genes such as *TNF, IL6*, and *CXCL11 *is observed in monocytes following tAVNS. Furthermore, a significantly decreased *IL6* and *TNF* signaling is observed between monocytes following tANS. In accordance with the transcriptomic finding, we observed a significant reduction in LPS-induced TNF levels in whole blood culture following tAVNS (Pre-10LPS vs. Post-10LPS: 2738.8 ± 470.3 pg/mL vs. 2056.5 ± 618.7 pg/mL; **P=0.0008).

Our findings reveal significant alterations in the transcriptomic landscape of PBMCs, particularly in monocyte subsets following tANS, both in the presence and absence of an inflammatory LPS challenge. These studies provide the first evidence for tANS-induced alterations in circulating immune cells. Our data will be broadly useful to further explore the changes in immune responses induced by tANS.

Keywords: Inflammation, Transcutaneous Auricular Nerve Stimulation (tANS), scRNAseq

## **P20** Trigeminal nerve stimulation as a novel approach for non-electrophilic NRF2 activation

### Keren Powell^1,2^, Steven Wadolowski^1,2^, Willians Tambo^1,2,3^, Julia Jinu^1,4^, Chunyan Li^1,2,3,5^

#### ^1^Translational Brain Research Laboratory, The Feinstein Institutes for Medical Research, Manhasset, NY, USA; ^2^Institute of Bioelectronic Medicine, The Feinstein Institutes for Medical Research, Manhasset, NY, USA; ^3^Elmezzi Graduate School of Molecular Medicine at Northwell Health, Manhasset, NY, USA; ^4^Biology Department, Adelphi University, Garden City, NY, USA; ^5^Donald and Barbara Zucker School of Medicine at Hofstra/Northwell, Hempstead, NY, USA

##### **Correspondence:** Keren Powell (Kpowell3@northwell.edu); Chunyan Li (Cli11@northwell.edu)

*Bioelectronic Medicine 2025,* **11(1):**P20

Background: Nuclear factor erythroid 2-related factor 2 (NRF2) is a key transcription factor that regulates cellular defense mechanisms against oxidative stress by controlling the expression of antioxidant molecules. Given its central role in redox homeostasis, NRF2 has emerged as a promising therapeutic target. However, the clinical application of pharmacological NRF2 activators is often constrained by their electrophilic nature, which may pose safety concerns due to excessive electrophilic reactivity. Recent studies have highlighted the potential of bioelectronic stimulation techniques—such as vagus, transcranial, facial, and median nerve stimulation—as alternative strategies for NRF2 activation, though the underlying mechanisms, particularly whether they involve electrophilic or non-electrophilic pathways, remain poorly understood. Notably, the effect of trigeminal nerve stimulation (TNS) on NRF2 activation has yet to be explored. Previous evidence suggests that TNS induces the release of endogenous calcitonin gene-related peptide (CGRP), a neuropeptide with potent antioxidant properties and a potential role in non-electrophilic NRF2 activation. Investigating the impact of TNS on NRF2 signaling could therefore provide novel insights into non-electrophilic approaches for enhancing cellular resilience against oxidative stress.

Methods: Thirty-six male Sprague-Dawley rats were randomly assigned to three groups: sham, TNS, and TNS with CGRP antagonist treatment. The TNS and TNS with CGRP antagonist groups received intermittent electronic TNS for 1 hour (20 seconds on, every 5 minutes). Brain tissues were collected immediately following the stimulation and analyzed for CGRP, CGRP receptor expression, nuclear and cytoplasmic NRF2, phosphorylated NRF2, and NRF2-activated oxidative stress-related genes. The inclusion of the CGRP antagonist-treated group allowed for the assessment of CGRP as a principal mediator and its role in NRF2 activation, downstream antioxidant gene expression, and associated signaling pathways.

Results: TNS significantly enhanced NRF2 activation, as demonstrated by a twofold increase in phosphorylated NRF2 and a 1.7-fold increase in nuclear NRF2 levels. This activation was accompanied by a substantial upregulation of NRF2-mediated antioxidative gene transcription, increased levels of reduced glutathione, and the preservation of lipid peroxidation homeostasis, suggesting a non-electrophilic activation mechanism. Further supporting this notion, TNS induced the upregulation of p62, SIRT1, and AMPK, indicating potential involvement of non-electrophilic signaling pathways. These effects were mediated by a twofold increase in CGRP expression and a 1.7-fold increase in CGRP receptor expression, as pharmacological blockade of CGRP signaling markedly attenuated these responses.

Conclusions: These findings indicate that TNS-induced NRF2 activation, mediated through the precise modulation of CGRP, may represent a viable therapeutic strategy for oxidative stress-related diseases. This approach offers potential advantages over conventional electrophilic activators by mitigating additional cellular stress and providing a controlled, non-electrophilic mechanism for NRF2 upregulation.

Keywords: NRF2; trigeminal nerve stimulation; CGRP; non-electrophilic

## **P21** Trigeminal nerve stimulation-induced oxygen-conserving reflex enhances cognitive function by restoring neuropeptide signaling in VCID

### Willians Tambo^1,2,3^, Keren Powell^1,2^, Steven Wadolowski^1,2^, Christopher LeDoux^1,4^, Chunyan Li^1,2,3,5^

#### ^1^Translational Brain Research Laboratory, The Feinstein Institutes for Medical Research, Manhasset, NY, USA; ^2^Institute of Bioelectronic Medicine, The Feinstein Institutes for Medical Research, Manhasset, NY, USA; ^3^Elmezzi Graduate School of Molecular Medicine at Northwell Health, Manhasset, NY, USA; ^4^Department of Biology, Hofstra University, Hempstead, NY, USA; ^5^Donald and Barbara Zucker School of Medicine at Hofstra/Northwell, Hempstead, NY, USA

##### **Correspondence:** Willians Tambo (wtamboayol@northwell.edu); Chunyan Li (cli11@northwell.edu)

*Bioelectronic Medicine 2025,* **11(1):**P21

Introduction: Vascular cognitive impairment and dementia (VCID) is the second most prevalent form of dementia, and currently, no effective treatment exists. Chronic cerebral hypoperfusion (CCH), a primary contributor to VCID pathogenesis, induces a cascade of pathological events, particularly affecting the microvasculature, ultimately leading to cognitive decline. Although pharmacological treatments for VCID have shown some promise, no targeted therapies without systemic side effects have been developed. Bioelectronic medicine, particularly trigeminal nerve stimulation (TNS), has gained attention as a potential therapeutic strategy for both acute and chronic neurological conditions. In cases of acute ischemic brain disorders, TNS has been shown to improve cerebral perfusion, modulate microvascular function, reduce blood-brain barrier (BBB) permeability, and protect the cerebrovascular endothelium. These mechanisms suggest that TNS may be an effective treatment option for neurological disorders characterized by cerebral hypoperfusion, such as CCH-induced VCID.

Objective: This study aims to evaluate the therapeutic effects of TNS on microvascular dysfunction and cognitive decline associated with CCH, a hallmark of VCID.

Method: Forty-eight male Sprague-Dawley rats were subjected to CCH through bilateral vessel occlusion (2VO), followed by evaluation of vascular, non-vascular, and cognitive function markers at 2, 4, and 6 weeks post-2VO, representing different severities of VCID. Trigeminal nerve stimulation parameters were optimized to trigger the oxygen-conserving reflex, a potent endogenous mechanism that supports survival in hypoxic and ischemic environments. Intermittent TNS was applied for 30 minutes per session, with 20-second stimulation intervals every 5 minutes. The stimulation regimen began 3 days after 2VO and was administered 5 days per week for a total duration of 6 weeks.

Results: CCH leads to progressive microvascular collapse and vascular degeneration in the hippocampus, driving cognitive dysfunction. This stepwise microvascular dysfunction, particularly vascular collapse, is strongly correlated with the gradual decline in cognitive abilities. CCH disrupts BBB integrity, promotes coagulation, and induces oxidative stress and vascular inflammation, all of which contribute to the primary microvascular collapse. TNS-induced oxygen-conserving reflex significantly mitigates hippocampal microvascular collapse, associated with increased levels of calcitonin gene-related peptide (CGRP) and decreased levels of endothelin-1 and fibrinogen. Notably, TNS therapy enhances hippocampal angiogenesis and restores BBB integrity in the CCH model. The improvement in microvascular integrity facilitated by TNS corresponds with a marked enhancement in cognitive performance observed during the later stages of CCH.

Conclusion: TNS exerts a significant effect on the microvasculature, highlighting its potential as a therapeutic strategy for VCID. By modulating cerebrovascular function, TNS addresses microvascular dysfunction, potentially improving cerebral blood flow and cognitive outcomes. Its non-invasive nature and targeted impact on microvascular integrity make TNS a promising intervention for VCID, meriting further clinical investigation.

Keywords: vascular dementia, microvascular, CGRP, neuromodulation

## **P22** Level-dependent separation of sensory and motor fascicles in the human cervical vagus nerve

### Naveen Jayaprakash^1^, Nicole Carpentiere^1^, Khaled Qanud^1^, Nafiseh Saleknezhad^1^, Siyar Bahadir^1^, Ibrahim Mughrabi^1^, Lucas Cang^1^, Todd Levy^1^, Avantika Vardhan^1^, Viktor Toth^1^, Tara Yari^2^, Zeinab Nassrallah^2^, Mary Barbe^3^, Larry Miller^1^, Theodoros Zanos^1^, Stavros Zanos^1,2,4^

#### ^1^Institute of Bioelectronic Medicine, Feinstein Institutes for Medical Research, Manhasset, NY, USA; ^2^Zucker School of Medicine at Hofstra/Northwell, Hempstead, NY, USA; ^3^Lewis Katz School of Medicine, Temple University, Philadelphia, PA, USA; ^4^Elmezzi Graduate School of Molecular Medicine, Manhasset, NY, USA

##### **Correspondence:** Naveen Jayaprakash (njayaprakash@northwell.edu); Stavros Zanos (szanos@northwell.edu)

*Bioelectronic Medicine 2025,* **11(1):**P22

Vagus nerve stimulation (VNS) is emerging as a potential treatment of disorders associated with chronic inflammation. To reduce side-effects and improve the precision of VNS, it may be useful to target specifically sensory or motor fascicles in the cervical VN with spatially selective VNS. The extent to which sensory and motor fascicles are separated in the cervical VN is unknown.

Six vagus nerves were extracted from 3 embalmed cadavers, from the nodose ganglion to the subdiaphragmatic branches. Each nerve was scanned using micro-CT; 3D reconstruction of nerve fascicle trajectories was performed using Slicer software. IHC was performed in the same nerves, with stains for neurofilament (NF), myelin basic protein (MBP), choline acetyltransferase (ChAT), and tyrosine hydroxylase (TH). Based on these markers, fibers were categorized as motor fibers (NF+, MBP+, ChAT+/TH+) or sensory fibers (NF+, MBP-, ChAT-, TH-). To identify unmyelinated sensory fibers, we performed Peripherin (P+) staining; P+ fibers are NF+ but TH- and ChAT-. In the micro-CT images, fascicles emerging caudally from the nodose ganglion were classified as sensory, and fascicles bypassing the nodose as motor. To validate the classification, we quantified counts of sensory and motor fibers from IHC images of the same fascicles.

We found that fascicles that were classified as sensory (in micro-CT) had significantly more sensory fibers (in IHC) than motor fascicles. Additionally, fascicles rich in TH were low in ChAT, and fascicles rich in ChAT were low in TH. Longitudinal tracking of fascicles in micro-CT images showed that motor and sensory fascicles merge at different levels, which vary between individuals and between the left and right vagus nerves. Almost complete mixing of sensory and motor fascicles was observed at the mid-cervical level, approximately 3 to 6 cm from the center of the nodose ganglion (level at which the ganglion's diameter is the greatest). Additionally, at least one motor fascicle in each nerve remained separate from the sensory or mixed fascicles, throughout the length of the cervical VN. Fiber count distributions within fascicles agree with the micro-CT findings of initial sensory-motor separation and progressive mixing.

Our findings indicate that sensory-motor fascicle separation is maintained in the upper and mid-cervical cervical VN in humans. Placement of VNS devices at or above the mid-cervical level in principle permits spatially selective activation of sensory or motor functions of the VN.

Keywords: Vagus nerve stimulation, Neuromodulation , Micro-CT , Immunohistology

## **P23** Machine learning model for identifying PTSD presence and severity using multimodal physiological data: toward scalable and accessible diagnostic tools

### Fylaktis Fylaktou^1,2,3,4^, Shubham Debnath^1,2,3^, Pooja Shaam^1,5,6^, Laura Ryniker^1,5,6^, Rebecca M. Schwartz^1,2,5,6^, Theodoros P. Zanos^1,2,3,4^

#### ^1^Northwell Health, New Hyde Park, NY, USA; ^2^Donald and Barbara Zucker School of Medicine at Hofstra/Northwell, Hempstead, NY, USA; ^3^Institute of Health Systems Science, Feinstein Institutes for Medical Research, Northwell Health, Manhasset, NY, USA; ^4^Institute of Bioelectronic Medicine, Feinstein Institutes for Medical Research/Northwell Health, Manhasset, NY, USA; ^5^Department of Occupational Medicine, Epidemiology and Prevention, Donald and Barbara Zucker School of Medicine at Hofstra/Northwell, Hempstead, NY, USA; ^6^Center for Traumatic Stress, Resilience and Recovery at Northwell Health, Great Neck, NY, USA

##### **Correspondence:** Fylaktis Fylaktou (ffylaktou@northwell.edu); Theodoros P. Zanos (tzanos@northwell.edu)

*Bioelectronic Medicine 2025,* **11(1):**P23

Post-traumatic stress disorder (PTSD) is a complex mental health condition with significant societal and individual burdens. Current diagnostic methods rely heavily on subjective self-reports and clinical interviews, which can be influenced by biases and lack objective physiological markers. To address this gap, we developed a machine learning model capable of identifying PTSD presence and severity using multimodal physiological data, including electroencephalogram (EEG), electrocardiogram (ECG), and beat-to-beat blood pressure (BBBP) derived features. Participants underwent controlled tests designed to drive their autonomic nervous system into either sympathetic or parasympathetic states, providing dynamic physiological responses for analysis.

The study included 79 participants (30 PTSD patients and 49 healthy individuals), who completed 4 tasks (Squat test, Valsalva Maneuver, Deep Breathing, Diving Reflex). Physiological signals were recorded during these tasks and at baseline, and features were extracted from EEG, ECG, and BBBP. These features were used to train and validate machine learning models, including classification models for PTSD presence and regression models for severity prediction. Model performance was evaluated through stratified 5-fold cross-validation and assessed using Area under the Curve of the Receiver Operating Curve (ROC-AUC) and area under the Precision-Recall curve (AUC-PR).

The model demonstrated strong performance in classifying PTSD presence and severity, achieving a testing ROC-AUC of 93% and an AUC-PR of 91%. Notably, the integration of multimodal data (EEG, ECG, and blood pressure) significantly improved performance compared to reduced-modality approaches. The regression models for severity prediction with all modalities for testing data also showed promising results, with an R² value of 0.78 indicating a strong correlation between predicted and actual severity scores, which performed better than the alternatives. To enhance clinical applicability, we further investigated whether the model’s performance could be maintained using fewer modalities or baseline recordings alone. Results indicated that while the full multimodal approach provided the highest ROC-AUC (93%), the model that used all modalities and baseline data still yielded promising results, with accuracy dropping only marginally to 92%. Reduced modality models, while less informative than task-driven data, also showed potential for PTSD identification.

These findings highlight the feasibility of using machine learning and physiological data for objective PTSD assessment. The ability to identify PTSD with reduced modalities or baseline recordings suggests potential for developing simplified, scalable diagnostic tools that could be deployed in clinical or remote settings. However, limitations include limited sample size, generalizability to diverse populations, and the need for further validation in real-world settings. Future work will focus on validating the model in larger, more diverse cohorts and exploring its applicability to other mental health conditions.

This study represents a significant step toward objective, physiology-based PTSD diagnosis, with implications for improving diagnostic accuracy, reducing reliance on subjective measures, and enhancing accessibility to mental health care.

Keywords: Post-traumatic stress disorder (PTSD), Electroencephalogram (EEG), Electrocardiogram (ECG), Beat-to-beat blood pressure (BBBP), Autonomic nervous system (ANS), Multimodal data fusion, Objective mental health diagnostics, Classification models, Regression models, Severity prediction, Clinical decision support systems

## **P24** First-in-human orbital flap reconstruction and radiation seed implantation for skull base meningioma

### Shayan Huda^1,2^, Marcio Yuri Ferreira^1^, Brianna Suffren^1^, Faina Ablyazova^1^, John A Boockvar^1^, David J Langer^1^, Netanel Ben-Shalom^1^

#### ^1^Department of Neurosurgery, Lenox Hill Hospital, Donald and Barbara Zucker School of Medicine at Hofstra/Northwell, New York, NY, USA; ^2^CUNY School of Medicine, New York, NY, USA

##### **Correspondence:** Shayan Huda (shuda@northwell.edu); Netanel Ben-Shalom (nbenshalom@northwell.edu)

*Bioelectronic Medicine 2025,* **11(1):**P24

Background: Recurrent skull base meningiomas involving the orbit and anterior cranial fossa pose significant surgical and therapeutic dilemmas due to their proximity to critical neurovascular structures, high recurrence rates, and the inherent complexities of re-irradiation. While radiation therapy remains a cornerstone of treatment, cumulative dose constraints in patients with prior radiation exposure often limit the feasibility of further external beam therapy. Conventional surgical approaches can reduce tumor burden; however, they may fail to address the extensive defects or provide sufficient local control in heavily pretreated fields. In this case report, we present a first-in-human technique that combines free tissue transfer for orbital and craniofacial reconstruction with intraoperative brachytherapy using cesium seeds. By delivering localized radiotherapy directly to the tumor bed, this approach aims to achieve improved local tumor control while mitigating the systemic and regional toxicity associated with wide-field re-irradiation.

Case Presentation: A 47-year-old female with a complex oncologic background—most notably childhood right-eye retinoblastoma managed with incomplete radiation and multiple prior resections of a right frontal anaplastic meningioma (WHO grade III)—presented with progressive enlargement of a right facial and anterior skull base mass. Magnetic resonance imaging revealed recurrent tumor encroaching upon the orbit, nasal cavity, and frontal fossa. Given the patient’s extensive surgical history and limited tolerance for additional radiation, a multidisciplinary team devised a novel approach: (1) wide surgical resection of the meningioma, including removal of involved orbital and skull base structures; (2) immediate intraoperative placement of cesium-131 seeds in the resection cavity for focal brachytherapy; and (3) orbital and craniofacial reconstruction using a left latissimus dorsi free flap alongside a mesh cranioplasty to restore the bony framework. Intraoperatively, the seeds were meticulously implanted to ensure uniform dose distribution, thus targeting microscopic residual disease. Postoperative care was complex, requiring prolonged intensive care monitoring. The patient experienced transient hydrocephalus necessitating an external ventricular drain, followed by eventual conversion to a permanent shunt. Despite these challenges, the latissimus dorsi free flap maintained robust perfusion with no evidence of partial or total flap failure. Serial postoperative imaging over several weeks revealed no early local tumor recurrence, supporting the efficacy of the combined surgical and brachytherapy strategy. Additionally, the flap provided satisfactory contour restoration and protective coverage of the orbital cavity.

Conclusion: This case illustrates a pioneering surgical paradigm that integrates aggressive tumor resection, free flap reconstruction for extensive skull base and orbital defects, and intraoperative cesium brachytherapy for precise, localized radiation delivery. The early clinical results suggest that direct seed implantation can enhance tumor control while mitigating the potential toxicities of broader re-irradiation fields. Looking ahead, more extensive studies with longer follow-up are essential to determine the durability of local control, flap viability, and overall impact on patient quality of life. Nonetheless, this combined approach holds promise as a viable treatment modality for patients with recurrent, anatomically challenging skull base meningiomas, particularly those with limited conventional radiation options.

Keywords: Recurrent Meningioma, Skull Base Surgery, Orbital Reconstruction, Brachytherapy, Cesium Seed Implantation

## **P25** Application of bedside cranial ultrasound in patients with a translucent cranial implants: efficacy and utilization of the “neurosurgical stethoscope”

### Shayan Huda^1,2^, Marcio Yuri Ferreira^1^, Omer Doron^3^, Brianna Suffren^1^, Faina Ablyazova^1^, Randy S D’Amico^1^, David J Langer^1^, Netanel Ben-Shalom^1^

#### ^1^Department of Neurosurgery, Lenox Hill Hospital, Donald and Barbara Zucker School of Medicine at Hofstra/Northwell, New York, NY, USA; ^2^CUNY School of Medicine, New York, NY, USA; ^3^Neuroendovascular Program, Massachusetts General Hospital & Brigham and Women’s Hospital, Harvard University, Boston, MA, USA

##### **Correspondence:** Shayan Huda (shuda@northwell.edu); Netanel Ben-Shalom (nbenshalom@northwell.edu)

*Bioelectronic Medicine 2025,* **11(1):**P25

Background: Brain imaging is central to the management of neurosurgical patients in the acute postoperative setting. Computed tomography (CT) is the gold standard for the postoperative surveillance and identification of life-threatening complications, including hematomas, hydrocephalus, and midline shift. However, it entails the transportation of often unstable patients to radiology suites and exposes them to ionizing radiation. These challenges can be particularly problematic in critical care environments. Ultrasound is a well-established modality in many medical fields due to its portability, lack of radiation, cost-effectiveness, and real-time capability. In neurosurgery, the US has demonstrated several invasive and non-invasive therapeutic and/or diagnostic applications, such as US-guided resection, neuromodulation for movement disorders, local drug delivery via blood-brain barrier disruption, and traumatic brain injury. Transcranial ultrasound is a noninvasive, real-time imaging modality that has traditionally been limited in postoperative neurosurgical patients due to skull impediments. However, the advent of translucent cranial implants (TCI) may provide an acoustic window, allowing for practical bedside transcranioplasty US (TCUS).

Objective: This case series assesses the feasibility and applicability of the TCUS in the postoperative evaluation of seven nonconsecutive patients with TCI. It also discusses the diagnostic concordance between TCUS images and computed tomography (CT) in the reported cases.

Methods: Following PROCESS 2023 guidelines, a retrospective review was conducted of seven patients (mean age 62 years, range 49–76) who underwent cranioplasty with TCI (ClearFit®, Longeviti Neuro Solutions LLC) after craniectomy to treat various pathologies. Each patient underwent bedside TCUS using a Butterfly iQ handheld probe prior to urgent CT for clinical concerns, in accordance with institutional protocol. Pathological and non-pathological TCUS findings were qualitatively compared with CT findings.

Results: In all seven patients, TCUS findings (e.g., ventricular size, graft patency via Doppler, catheter location, midline shift) were concordant with CT scans, and no additional hematomas or other critical findings were missed. The TCUS took under five minutes, minimized patient transport needs, and required minimal repositioning. Operator dependency and limited field of view were noted challenges.

Conclusion: TCUS allowed reliable bedside ultrasound evaluation for postoperative neurosurgical complications and demonstrated strong concordance with CT findings in this small case series. Although TCUS cannot replace CT at this stage, our findings demonstrate that it may serve as an promising “neurosurgical stethoscope” for rapid triage and monitoring in the postoperative setting. This is an initial report on the use of TCUS in our department and represents the first study assessing TCUS applicability as a diagnostic tool in the postoperative period after cranioplasty. Ongoing studies will allow us to establish sensitivity, specificity, broader clinical applicability and the comparative cost-effectiveness between TCUS and TC.

Keywords: Transcranial Ultrasound, Translucent Cranial Implants, Bedside Ultrasound, Postoperative Neurosurgical Evaluation

## **P27** Mitochondrial Transfer Therapy (MTT) a potential therapy for osteoarthritis

### Henintsoa Fanjaniaina Andriamifidy, Pooja Swami, Haixiang Liang, Kenneth R. Zaslav, Daniel A. Grande

#### Orthopaedic Research Laboratory, Feinstein Institutes for Medical Research, Northwell Health, Manhasset, NY, USA

##### **Correspondence:** Henintsoa Fanjaniaina Andriamifidy (hfanjaniai@northwell.edu); Daniel A. Grande (dgrande@northwell.edu)

*Bioelectronic Medicine 2025,* **11(1):**P27

Purpose: Mitochondria are cellular powerhouses and vital regulators of cellular homeostasis and tissue repair post-injury. Recent studies underscore mitochondria transfer as a key mechanism by which stem cells rescue cellular function. This study aims to explore how mitochondrial transfer therapy (MTT) from young synovial fluid-derived stem cells (SF-MSCs) can be a potential therapeutic for osteoarthritis (OA).

Methods: SF-MSCs from a 15-year-old donor (young SF-MSCs) were used to collect mitochondria for *in-vitro* studies. SF-MSCs from a 65-year-old patient (adult SF-MSCs) were co-cultured with mitochondria from young SF-MSCs under various conditions. Expression of Collagen II and Sirtuin I under normal and inflammatory conditions were evaluated through immunocytochemistry. Uptake of mitochondria was quantified after short time exposure to low dose of mitomycin C (MMC), an inducer of DNA damage and senescence.

Results: MTT from young SF-MSCs showed the capacity to improve cellular adaptation of adult SF-MSCs under stressful conditions. Adult SF-MSCs increased expression of Collagen II and Sirtiun I after MTT from young SF-MSCs in a dose dependent manner. After a dose of MMC, adult SF-MSCs demonstrated more uptake of mitochondria compared to control without MMC.

Conclusion: These findings suggest a therapeutic potential of MTT from young SF-MSCs donor by enhancement of an anabolic pathway related to cartilage repair. However, underlying cellular and molecular mechanisms warrant further research.

*Adult SF-MSCs increased expression of Collagen II without changing Sirtuin I expression after MTT from young SF-MSCs in normal conditions and in a dose dependent manner under inflammatory conditions.*

Keywords: mitochondria transfer, OA: osteoarthritis, synovial fluid stem cells

## **P28** Implantable bioelectronic device for gastric interfacing to study the stomach-brain axis in feeding behavior

### Rajib Mondal^1^, Atharva Sahasrabudhe^2^, Karen Pang^3^, Frank Kelley^4^, Polina Anikeeva^5^

#### ^1^Harvard-MIT Division for Health-Sciences and Technology, Research Laboratory for Electronics, K. Lisa Yang Brain-Body Center-MIT, Cambridge, MA, USA; ^2^Research Laboratory for Electronics-MIT, Cambridge, MA, USA; ^3^Brain and Cognitive Sciences at MIT, Research Laboratory for Electronics, K. Lisa Yang Brain-Body Center-MIT, Cambridge, MA, USA; ^4^Electrical Engineering and Computer Science Department at MIT, Research Laboratory for Electronics-MIT, Cambridge, MA, USA; ^5^Brain and Cognitive Sciences at MIT, Research Laboratory for Electronics, Department for Materials Sciences and Engineering, K. Lisa Yang Brain-Body Center-MIT, Cambridge, MA, USA

##### Correspondence: Rajib Mondal (rmondal@mit.edu); Polina Anikeeva (anikeeva@mit.edu)

*Bioelectronic Medicine 2025,* **11(1):**P28

Bioelectronic medicine holds a great promise to diagnose and treat various diseases of peripheral organs ranging from metabolic disorders to inflammatory diseases. This approach leverages bioelectronic devices to stimulate and record the body’s own electrical signals, most commonly neural activity, to alter the state of an organ. The design and development of implantable devices for peripheral organs is often challenging due to the delicateness and heterogeneity of peripheral tissue. In this poster we present a versatile and scalable approach to fabricate bioelectronic devices for the stomach. More specifically, we present a wirelessly controlled device that interfaces with the gastric serosa to stimulate stomach neurons using optogenetics. At the same time this device can record gastric electrophysiology (EGG) and temperature in chronic rodent studies to assess gastric organ physiology in real-time. The device is used to modulate feeding behavior and investigate gastric stimulation as a potential therapeutic strategy for obesity.

Keywords: Bioelectronic Medicine, gut-brain-axis, implantable devices, obesity

## **P29** IL-1β -responsive neurons in the bed nucleus of the stria terminalis mediate the acute stress responses via adrenergic signaling

### Okito Hashimoto^1^, Tyler Hepler^1^, Aisling Tynan^1^, Alejandro Torres^1^, Carlos E. Bravo-Iniguez^1^, Jian Hua Li^1^, Michael Brines^1^, Kevin J. Tracey^1,2,3^, Sangeeta S. Chavan^1,2,3^

#### ^1^Laboratory of Biomedical Sciences, Institute for Bioelectronic Medicine, Feinstein Institutes for Medical Research, Northwell Health, Manhasset, NY, USA; ^2^The Elmezzi Graduate School of Molecular Medicine, Manhasset, New York, USA; ^3^Donald and Barbara Zucker School of Medicine at Hofstra/Northwell, Hempstead, NY, USA

##### **Correspondence:** Okito Hashimoto (ohashimoto@northwell.edu); Sangeeta S. Chavan (schavan@northwell.edu)

*Bioelectronic Medicine 2025,* **11(1):**P29

The mechanistic pathways linking cytokine-mediated stress networks in the brain remain poorly understood. We discovered a specific population of neurons in the bed nucleus of the stria terminalis (BNST), which shape diverse responses to interleukin-1β (IL-1β)-mediated stress phenotype. Chemogenetic activation of IL-1β-captured neurons in the BNST is sufficient to recapitulate IL-1β-induced inflammatory responses. Psychological stress activates distinct neuronal ensembles in the BNST that are also active in response to IL-1β. Chemogenetic activation or selective ablation revealed that one of IL-1β-responsive neuronal subsets in the BNST is sufficient and necessary to broadly retrieve stress-induced inflammatory responses. Blocking of adrenergic signaling by β-blockers abrogates BNST-evoked responses. Given the variety of stress responses driven by the BNST- adrenergic signaling pathway, the BNST can be a potential target using bioelectronic devices for stress-related inflammatory disorders.

Keywords: BNST, IL-1β, acute stress, adrenergic signaling pathway

## **P30** The hippocampus and entorhinal cortex in the gut microbiome–brain axis

### Joshua J. Strohl, Joshua M. Glynn, Joseph C. Carrión, Patricio T. Huerta

#### Feinstein Institutes for Medical Research, Manhasset, NY, USA

##### **Correspondence:** Joshua J. Strohl (jstrohl@northwell.edu); Patricio T. Huerta (phuerta@northwell.edu)

*Bioelectronic Medicine 2025,* **11(1):**P30

Introduction: Oral antibiotic use, while crucial for combating infections, disrupts the gut microbiome and can negatively impact brain function. This study investigates the effects of gut microbiome depletion (induced by a broad-spectrum antibiotic cocktail) on the neural activity of the hippocampus and entorhinal cortex, two brain regions essential for spatial cognition. We also explore the potential of butyrate supplementation to mitigate the neurological consequences of antibiotic-induced microbiome disruption.

Methods: Male C57BL/6J mice received either an antibiotic cocktail (ABX: ampicillin, neomycin, gentamicin, metronidazole, and vancomycin) or untreated water (CON) for four weeks. Separate cohorts received butyrate supplementation, either alone, or with antibiotics. Spatial cognitive performance was assessed with the clockmaze task (PMC6568215). Electrode arrays were surgically implanted into the hippocampus and the medial entorhinal cortex to record from freely moving mice (PMC8609830). We examined the spatial firing properties of individual neurons and the rhythmic activity of local neuronal ensembles. Blood-brain barrier integrity was assessed using positron emission tomography with the [^11^C]-iso-aminobutyric acid (AIB) radiotracer, and with immunohistochemistry to stain for tight junction proteins.

Results: ABX treatment results in significantly impaired spatial cognition in the clockmaze task (ABX mice fail to use spatial strategies when compared to the CON group). Hippocampal neurons from ABX mice show abnormal place cell properties, such as significantly larger place field sizes, reduced spatial information, and a lowered ability to encode the animal’s position in space. Furthermore, entorhinal neurons from ABX mice have disrupted grid cell properties. Notably, spectral analysis of brain oscillations shows that, in ABX mice, the hippocampus has decreased spectral power (across multiple bands) whereas the entorhinal cortex displays increased spectral power. We also find that the integrity of the blood-brain barrier is compromised in the entorhinal cortex of ABX mice, as demonstrated by increased AIB uptake, and decreased expression of the tight junction proteins claudin-5 and occludin. Supplementation with butyrate attenuates the effects of ABX treatment, preserving spatial cognition, the neurophysiological properties of the hippocampus and entorhinal cortex, and blood-brain barrier integrity.

Conclusions: These findings clearly demonstrate that antibiotic-induced disruption of the gut microbiome is causally linked to dysfunction of the hippocampus and entorhinal cortex, leading to deficits in spatial cognition. Butyrate supplementation effectively mitigates these detrimental effects, suggesting its potential as a therapeutic strategy against the neurological consequences of antibiotic use. This study highlights the crucial interplay between the gut microbiome and brain health, underscoring the potential of microbiome-targeted interventions, such as butyrate supplementation, for preserving cognitive function during antibiotic treatment.

Keywords: Neurophysiology, Spatial Cognition, Grid Cells, Place Cells, Brain Oscillations, Antibiotics, Butyrate

## **P31** Investigating the efficacy of Reparel® compression sleeves in alleviating tendinopathy: an in vivo study

### Henintsoa Fanjaniaina A^1^, Haixiang Liang^1^, Pooja Swami^1^, Jorden Xavier ^3^,Daniel Grande^1^, Kenneth Zaslav^2^

#### ^1^Orthopedic Research Laboratory, Feinstein Institutes for Medical Research, Manhasset, NY, USA; ^2^Lenox Hill Hospital, Northwell Health, New York, NY, USA; ^3^Albert Einstein College of Medicine, Bronx, NY, USA

##### **Correspondence:** Henintsoa Fanjaniaina A (handriamifid@northwell.edu); Kenneth Zaslav (kzaslav@northwell.edu)

*Bioelectronic Medicine 2025,* **11(1):**P31

Introduction: The Reparel® Sleeve, a Class I device with nano-semiconductor fibers reflecting body heat at therapeutic wavelengths, aims to alleviate joint and tendon pain by accelerating tissue healing. This study evaluated its efficacy on Achilles tendinopathy in a rabbit model.

Methods: Twelve rabbits with collagenase-induced Achilles tendinopathy were divided into three groups (n=4 each): control sleeve without nano-semiconductors, Reparel® Sleeve design I, and Reparel® Sleeve design II. Calf circumference, thermal imaging, and histological analysis (Picrosirius red staining and collagen fiber dispersion quantification) were performed at days 14 and 28. An in-house MATLAB program was used to quantify the collagen fiber dispersion/alignment within the Achilles tendon samples.

Results: At 28 days, experimental groups showed reduced calf circumference (4.85cm, 4.6cm) compared to the control (5.2cm, p<0.05). Thermal radiation imaging to externally assess the temperature of the tendon showed a reduced heat radiation in the treatment groups. Histology revealed improved collagen organization and tissue proliferation/remodeling in both Reparel® groups at day 14. Collagen width dispersion differences lacked statistical significance. However, values were lower for experimental groups which indicate lower degree of disorganization of collagen fibers.

Discussion: The Reparel® Sleeve reduced swelling, temperature, and improved histological outcomes, aligning with clinical findings in osteoarthritis^1^. The nano-semiconductors may mimic low-level laser therapy, promoting ECM reorganization and healing. The control group's partial healing is attributed to the single collagenase injection, limiting the model's chronicity. Small sample size limited statistical power.

Conclusion: This study demonstrates the Reparel® Sleeve's potential to reduce inflammation and promote tissue healing in Achilles tendinopathy. Our findings show that the Reparel® Sleeve can induce histological changes that reduce inflammation and promote tissue healing.

Keywords: Photobiomodulation, pain management, tendinopathy, tissue repair

**References**

1. Elphingstone, Joseph W et al. “Bioactive Knee Sleeve for Osteoarthritis: A Small Cohort Study.” *Southern medical journal* vol. 115,10 (2022): 773-779.

## **P32** A novel biomimetic scaffold for tissue engineering constructs for repair of alar cartilage

### Pooja Swami^1^, Henintsoa Fanjaniaina^1^, Haixiang Liang^1^, Azhar Khan^2^, Daniel Grande^1^

#### ^1^Orthopaedic Research Laboratory, Feinstein Institutes for Medical Research, Northwell Health, Manhasset, NY, USA; ^2^Shoolini University of Biotechnology and Management Sciences, Solan, H.P, India

##### **Correspondence:** Pooja Swami (pswami@northwell.edu); Daniel Grande (dgrande@northwell.edu)

*Bioelectronic Medicine 2025,* **11(1):**P32

Introduction: Nasal alar cartilage defects, arising from trauma, disease, or surgery, create significant aesthetic and functional impairments, including nasal valve collapse and airway obstruction. Autologous cartilage grafts, while commonly used, are limited by donor site availability and morbidity. Synthetic implants risk infection and extrusion. Tissue engineering offers a promising alternative by combining biocompatible scaffolds with cells to generate functional cartilage. This study aims at comparing the chondrogenic effect of two different combination of biomaterial composition of a tissue engineered scaffold on nasal chondrocytes in-vitro.

Methods**:** A circular scaffold design was created using CAD software and translated into a 3D-printed negative mold using acrylonitrile butadiene styrene (ABS) plastic. Two hydrogels were formulated: alginate/chitosan (AC) and alginate/chitosan/type II collagen (CAC). Type II collagen was incorporated into CAC to mimic the native cartilage extracellular matrix. Hydrogels were compressed within the molds, frozen, and lyophilized to create porous scaffolds. Rabbit nasal septal chondrocytes were seeded onto the scaffolds and cultured for up to 6 weeks. Constructs were evaluated using immunohistochemistry (IHC) for markers of chondrogenesis (Sox9, Collagen II, Aggrecan).

Results: Scaffolds maintained structural integrity and shape throughout the culture period. IHC analysis demonstrated collagen II deposition within both AC and CAC scaffolds.

The graphs represent the number of positively stained cells in both AC (left) and CAC (right) scaffolds. The images demonstrate positive IHC staining for A) Sox9, B) Collagen II , C) Aggrecan. Positive staining for Sox9, Collagen II, and Aggrecan indicated native chondrocyte phenotype maintained within both scaffold types (Fig. 1).

Discussion: This study demonstrates the feasibility of combining different biomaterials (AC, CAC) for tissue engineering tunable constructs as a surrogate for chondrogenic differentiation. This finding show that scaffold composition can be modified to provide a cellular environment that may facilitate cell-to-cell interaction and motivate target cells to adopt a more native phenotype. While both scaffold composition supported chondrocyte function, further research is needed to optimize ideal composition and evaluate the biomechanical properties of the engineered cartilage.

Conclusion: This study highlights the potential of combining different biomaterials for tissue engineering constructs for alar cartilage grafts. Future studies will focus on scaffold optimization, biomechanical evaluation, and in vivo testing to translate these findings into clinical practice.

Keywords: Chondrocyte, biomaterials, Scaffolds, Alar cartilage

**Fig. 1 (abstract P32).**
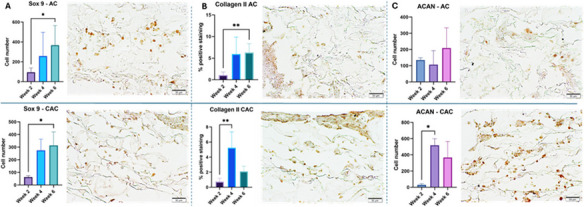
See text for description

## **P33** Autonomic, cardiac, and neural effects from non-invasive cervical vagus nerve stimulation

### Shubham Debnath^1,2^, Fylaktis Fylaktou^1,2,3^, Blake Gurfein^4^, Theodoros P. Zanos^1,2,3^

#### ^1^Institute of Bioelectronic Medicine, Feinstein Institutes for Medical Research, Manhasset, NY, USA; ^2^Institute of Health System Science, Feinstein Institutes for Medical Research, Manhasset, NY, USA; ^3^Donald and Barbara Zucker School of Medicine at Hofstra/Northwell, Northwell Health, Hempstead, NY, USA; ^4^Tivic Health, Fremont, CA, USA

##### **Correspondence:** Shubham Debnath (sdebnath@northwell.edu); Theodoros P. Zanos (tzanos@northwell.edu)

*Bioelectronic Medicine 2025,* **11(1):**P33

The vagus nerve is a key conduit in the autonomic nervous system and is involved with responses in cardiovascular, pulmonary, gastrointestinal, renal, hepatic, endocrine, and neural systems (Pavlov et al., 2018). While the use of implanted vagus nerve stimulation (VNS) devices is approved and has been used as an alternative treatment for drug-resistant epilepsy (Ben-Menachem, 2002), depression (Austelle et al., 2022), headache (Yuan & Silberstein, 2015), and as a supplement to stroke recovery rehabilitation (Dawson et al., 2021), there are inherent risks, complications, and costs related to surgical procedure and device operation (Ben-Menachem et al., 2014). Recent work has shown that non-invasive methods offer a means to stimulate the nerve without surgical intervention, and targeting auricular and cervical branches of the nerve have led to therapeutic responses on clinical populations. However, the mechanism and autonomic nervous system (ANS) responses are not well understood. In this work, non-invasive cervical VNS (ncVNS) was applied to healthy participants while recording physiological measurements to assess effects on the ANS, cardiac function, and brain activity, as well as how these responses fluctuate by varying stimulation parameters.

The ANS regulates and integrates bodily functions and internal organs, including control of the heart, blood vessels, and pupils. Instead of monitoring ANS activity by invasive methods on relevant neural targets, ANS-dependent physiological signals were recorded through accepted, clinical recording modalities, including electrocardiogram (ECG), beat-to-beat noninvasive blood pressure (NIBP), electroencephalography (EEG), and pupillometry. From raw ECG signals, heart rate variability (HRV) measures can be derived, particularly the root mean square of successive R-R interval differences (RMSSD) as a reliable index for parasympathetic activity down to 30 second intervals (Salahuddin et al., 2007).

Vitals measurements were taken before, during, and after ncVNS treatment of 20 healthy individuals. Compared to baseline measurement, the intervention resulted in a 97% increase in RMSSD and sustained pupil constriction, both outcomes linked to parasympathetic nervous system activation (Bertsch et al., 2012, McDougal & Gamlin, 2015). Additionally, EEG measurements showed an increase in frontal theta power by 24% and reduced gamma power in multiple brain regions, including a 66% decrease in the frontal lobe. These central nervous system effects are consistent with reduced anxiety and arousal.

Varying stimulation parameters, notably frequency and amplitude, also affected RMSSD in healthy participants. Four frequencies were tested at a supra-sensory threshold amplitude. While no single frequency caused a significantly greater response, each participant showed a preferred stimulation frequency; preferred frequencies led to an average of 40% increase in RMSSD, compared to 10% or less for each tested frequency. At each participant’s preferred frequency, three amplitudes were tested at sub-sensory, sensory, and supra-sensory threshold; RMSSD and other recorded vitals effects were reproduced at supra-sensory threshold amplitude, with smaller or no effect at the lower amplitudes.

While these results were in a small sample of healthy, young individuals, the results suggest that ncVNS approaches may have clinical utility in various patient populations, including post-traumatic stress disorder (PTSD), epilepsy, ischemic stroke recovery, and cardiac disorders. Future steps to this work will include varying additional stimulation parameters for patient or patient population optimization, as well as examining feasibility and efficacy in larger, more diverse populations.

Keywords: noninvasive, vagus nerve stimulation, heart rate variability, autonomic nervous system, clinical recording

## **P34** Wearable disposable electrotherapy for accelerated skin wound healing

### Mojtaba Belali Koochesfahani, Mohamad FallahRad, Kyle Donnery, Rayyan Bhuiyan, Miguel Posada, Jana M Elwassif, Baseer Mirza, Niranjan Khadka, Marom Bikson

#### Biomedical Department of City College of New York City University of New York, NY, USA

*Bioelectronic Medicine 2025,* **11(1):**P34

Electrical stimulation is established to accelerating wound healing. This study presents the development and validation of a novel Wearable Disposable Electrotherapy (WDE) device designed to deliver low-intensity direct current stimulation to full-thickness wounds. The device integrates a battery cell, stimulation hydrogel electrodes embedded in a non-woven wound dressing pad, and a skin adhesive interface resembling a conventional bandage for ease of application.

Battery sizing was guided by isotemporal trajectory theory, while finite element method (FEM) simulations predicted a uniform current density (~0.6 A/m²) across the wound area. The efficacy of the WDE device was evaluated in a rodent model using eight male Sprague Dawley rats (7 to 9 weeks old, 250-300 g). Under isoflurane anesthesia, one full-thickness dorsal excision (10 mm diameter) was created. A standardized wound care protocol ensured reproducibility. Electrical stimulation 250 ± 50 μA was applied for 120 minutes daily over 14 days, with electrodes positioned bilaterally across each wound. Control animals received sham bandages without stimulation. Wounds were protected with a non-woven dressing and a custom vest made of self- adhesive bandages to ensure stable electrode placement. Wound area reduction was monitored. Preliminary results indicate that wounds treated with the WDE device closed significantly faster (more than 25%) compared to the sham group (β = −0.011, SE = 0.004, z = −2.845, p = 0.004), confirming the device's ability to enhance wound healing. The WDE device effectively maintained electrode-skin contact and delivered consistent stimulation throughout the experimental period, demonstrating its practical application as a wearable therapeutic device.

Keywords: Electrical stimulation, Wound healing, Wearable electrotherapy, Direct current stimulation

## **P35** Imaging-based network biomarker enables prospective identification of placebo responders across therapeutic modalities

### János A Barbero, An Vo, Yilong Ma, Shichun Peng, Chris C Tang, David Eidelberg

#### Center for Neurosciences, The Feinstein Institutes for Medical Research, Manhasset, NY

##### **Correspondence:** János A Barbero (jbarbero@northwell.edu); David Eidelberg (deidelberg@northwell.edu)

*Bioelectronic Medicine 2025,* **11(1):**P35

Introduction: Placebo effects represent a significant challenge in Parkinson's disease (PD) trials, particularly in neurosurgical interventions, where up to 42% of patients show >50% improvement in motor ratings compared to 16% in medical trials (Goetz et al, 2008). We previously identified a sham surgery-related pattern (SSRP) from FDG-PET data in a trial of subthalamic gene therapy for advanced PD patients (Niethammer et al, 2018) using Ordinal Trends/Canonical Variates Analysis (OrT/CVA; Habeck et al, 2005). We found that baseline, pre-randomization SSRP expression predicted clinical improvement in the sham surgery arm (n=21, 6-month follow-up), but the generalizability of these results required further validation across different therapeutic contexts.

Methods: We analyzed data from three independent cohorts: the oral placebo arm (n=14, 1-month follow-up) of a double-blind phase I trial of nicotinamide riboside (NR) for de novo drug-naïve PD patients (Brakedal et al, 2022), the sham surgery cohort from our original study (n=21), and the injected placebo cohort (n=28, 9-month follow-up) from a double-blind phase II study of exenatide in PD (NCT04305002). We calculated SSRP expression in FDG-PET scans from each trial and standardized them to age- and sex-matched healthy controls (n=15). Resting-state fMRI scans were obtained in the NR study, in which we analyzed SSRP expression using amplitude of low-frequency fluctuations (ALff). Finally, we compared functional connectivity across timepoints in each trial to healthy controls.

Results: Pre-randomization SSRP expression predicted subsequent motor improvement across all three cohorts (sham surgery: r=0.46, p=0.036; oral placebo: r=0.58, p=0.030; injectable placebo: r=0.40, p=0.035), despite marked differences in intervention type, disease stage, and trial duration. The consistency of the predictive relationship across studies enabled pooling of the data, yielding a robust combined model (r=0.54, p<1E-5, n=63). SSRP expression in baseline ALff maps similarly predicted placebo responses (r=0.54, p=0.045). Under placebo conditions, the SSRP network gained significant functional connections between limbic regions (cingulate, amygdala) and motor areas (putamen, thalamus, cerebellar vermis). Notably, these connectivity changes reverted upon unblinding in the surgical cohort, where post-unblinding scans were taken.

Conclusion: The SSRP represents an intriguing network biomarker that predicts placebo susceptibility across markedly different placebo modalities. Its validation in non-invasive resting-state fMRI suggests potential clinical applicability. Incorporating baseline SSRP assessment into early-phase clinical trial design could enable stratification of placebo-susceptible individuals, potentially reducing the number of patients exposed to sham procedures while increasing statistical power.

Keywords: Parkinson’s disease, neuroimaging, FDG-PET, placebo

**References**

1. Goetz, Christopher G., Joanne Wuu, Michael P. McDermott, Charles H. Adler, Stanley Fahn, Curt R. Freed, Robert A. Hauser, et al. 2008. “Placebo Response in Parkinson’s Disease: Comparisons among 11 Trials Covering Medical and Surgical Interventions.” *Movement Disorders* 23 (5): 690–99. 10.1002/mds.21894.
2. Ko, Ji Hyun, Andrew Feigin, Paul J. Mattis, Chris C. Tang, Yilong Ma, Vijay Dhawan, Matthew J. During, Michael G. Kaplitt, and David Eidelberg. 2014. “Network Modulation Following Sham Surgery in Parkinson’s Disease.” *The Journal of Clinical Investigation* 124 (8): 3656–66. 10.1172/JCI75073.
3. Habeck, Christian, John W. Krakauer, Claude Ghez, Harold A. Sackeim, David Eidelberg, Yaakov Stern, and James R. Moeller. 2005. “A New Approach to Spatial Covariance Modeling of Functional Brain Imaging Data: Ordinal Trend Analysis.” *Neural Computation* 17 (7): 1602–45. 10.1162/0899766053723023.
4. Niethammer, Martin, Chris C. Tang, An Vo, Nha Nguyen, Phoebe Spetsieris, Vijay Dhawan, Yilong Ma, et al. 2018. “Gene Therapy Reduces Parkinson’s Disease Symptoms by Reorganizing Functional Brain Connectivity.” *Science Translational Medicine* 10 (469): eaau0713. 10.1126/scitranslmed.aau0713.
5. Brakedal, Brage, Christian Dölle, Frank Riemer, Yilong Ma, Gonzalo S. Nido, Geir Olve Skeie, Alexander R. Craven, et al. 2022. “The NADPARK Study: A Randomized Phase I Trial of Nicotinamide Riboside Supplementation in Parkinson’s Disease.” *Cell Metabolism* 34 (3): 396-407.e6. 10.1016/j.cmet.2022.02.001.

## **P36** Design and execution of a non-significant risk trial in the clinical assessment of an injectable neuromodulation electrode

### Manfred Franke^1^, Derrick Liu^1^, David Valencia^1^, Morgan McGaughey^1^, Victoria R Miduri^1^, Hesham Elsharkawy^1,2^, Krishnan Chakravarthy^1,3^, Stephan Nieuwoudt^1^, Amol Soin^1,4^

#### ^1^Neuronoff, Inc., Cleveland, OH, USA; ^2^Department of Anesthesiology, Pain and Healing Center, MetroHealth, Cleveland, OH, USA; ^3^Innovative Pain Treatment Solutions, Temecula, CA, USA; ^4^Ohio Pain Clinic, Dayton, OH, USA

##### **Correspondence:** Manfred Franke (manfred@neuronoff.com); Amol Soin (drsoin@soinneuroscience.com, drsoin@gmail.com)

*Bioelectronic Medicine 2025,* **11(1):**P36

Research Problem: Implanted neuromodulation devices are an emerging treatment alternative to opioids for chronic pain. However, neuromodulation remains a “last resort” due to the invasiveness of device implantation, maintenance, and removal. We designed an injectable electrode device to address the invasiveness of neuromodulation devices. Medical devices require rigorous, staged preclinical and clinical testing prior to regulatory approval and distribution. Here we present briefly 1) the preclinical testing considered in approval of a non-significant risk (NSR) clinical trial, and 2) NSR safety and efficacy data of the injectable electrode onto medial branch nerves of healthy human subjects.

Methods: Four Yorkshire pigs were injected with 2-3 electrodes onto each tibial nerve, with nerve activation defined by dewclaw flexion after stimulation (0-28 days). An NSR study (NCT06206356) using preclinically defined parameters was IRB approved. Ten healthy adult volunteers received 1-2 electrodes injected onto lumbar medial branch nerves for 28 days. Procedural safety and stimulation thresholds for subcutaneous afferents and multifidus musculature were evaluated and confirmed via ultrasound, physician, and patient-reported sensation.

Results: There were no procedure or device-related adverse events during porcine evaluation. After device placement, dewclaw flexion in pigs was achieved at 19.0±11.8 volts (V). At 28 days post-implantation, dewclaw flexion was achieved at 26.5±10.5 V. There were no serious adverse events reported during the NSR study. Intraoperative stimulation achieved multifidus muscle activation at 7.2 ± 1.9 V. Following 25 (±3) days, stimulation thresholds were substantially equivalent to day 0 intraoperative thresholds at 7.8 ± 1.3 V, indicative of electrode stability.

Significance: Successful electrode placement, stimulation, and removal in preclinical and NSR clinical trial settings supports the safety and effectiveness of the injectable electrode for neuromodulation.

Keywords: PNS, pain, peripheral nerve stimulation, neuromodulation, leads, nerve 

